# Neutrophils Recruited by NKX2‐1 Suppression via Activation of CXCLs/CXCR2 Axis Promote Lung Adenocarcinoma Progression

**DOI:** 10.1002/advs.202400370

**Published:** 2024-08-07

**Authors:** Anita S La'ah, Ping‐Hsing Tsai, Aliaksandr A. Yarmishyn, Lo‐Jei Ching, Chih‐Ying Chen, Yueh Chien, Jerry Chieh‐Yu Chen, Ming‐Long Tsai, Yi‐Chen Chen, Chun Ma, Po‐Kuei Hsu, Yung‐Hung Luo, Yuh‐Min Chen, Guang‐Yuh Chiou, Kai‐Hsi Lu, Wen‐Chang Lin, Yu‐Ting Chou, Mong‐Lien Wang, Shih‐Hwa Chiou

**Affiliations:** ^1^ Taiwan International Graduate Program in Molecular Medicine National Yang Ming Chiao Tung University and Academia Sinica Taipei 115 Taiwan; ^2^ Department of Medical Research Taipei Veterans General Hospital Taipei 112 Taiwan; ^3^ Institute of Pharmacology School of Medicine National Yang Ming Chiao Tung University Taipei 112 Taiwan; ^4^ Institute of Clinical Medicine National Yang Ming Chiao Tung University Taipei 112 Taiwan; ^5^ School of Medicine National Yang Ming Chiao Tung University Taipei 112 Taiwan; ^6^ Department of Surgery Taipei Veterans General Hospital Taipei 112 Taiwan; ^7^ Department of Chest Medicine Taipei Veterans General Hospital Taipei 112 Taiwan; ^8^ Taipei Cancer Center Taipei Medical University Taipei 110 Taiwan; ^9^ Department of Biological Science and Technology National Yang Ming Chiao Tung University HsinChu 300093 Taiwan; ^10^ Department of Medical Research and Education Cheng‐Hsin General Hospital Taipei 112 Taiwan; ^11^ Institute of Biomedical Sciences Academia Sinica Taipei 115 Taiwan; ^12^ Institute of Biotechnology National Tsing Hua University Hsinchu 300044 Taiwan; ^13^ Institute of Food Safety and Health Risk Assessment School of Pharmaceutical Sciences National Yang Ming Chiao Tung University Taipei 112 Taiwan; ^14^ Genomic Research Center Academia Sinica Taipei 115 Taiwan

**Keywords:** cheomkine, lung adenocarccinoma, single cell NGS, tumor microenvironment

## Abstract

NK2 Homeobox 1 (NKX2‐1) is a well‐characterized pathological marker that delineates lung adenocarcinoma (LUAD) progression. The advancement of LUAD is influenced by the immune tumor microenvironment through paracrine signaling. However, the involvement of NKX2‐1 in modeling the tumor immune microenvironment is still unclear. Here, the downregulation of NKX2‐1 is observed in high‐grade LUAD. Meanwhile, single‐cell RNA sequencing and Visium in situ capturing profiling revealed the recruitment and infiltration of neutrophils in orthotopic syngeneic tumors exhibiting strong cell‐cell communication through the activation of CXCLs/CXCR2 signaling. The depletion of NKX2‐1 triggered the expression and secretion of CXCL1, CXCL2, CXCL3, and CXCL5 in LUAD cells. Chemokine secretion is analyzed by chemokine array and validated by qRT‐PCR. ATAC‐seq revealed the restrictive regulation of NKX2‐1 on the promoters of *CXCL1*, *CXCL2*, and *CXCL5* genes. This phenomenon led to increased tumor growth, and conversely, tumor growth decreased when inhibited by the CXCR2 antagonist SB225002. This study unveils how NKX2‐1 modulates the infiltration of tumor‐promoting neutrophils by inhibiting CXCLs/CXCR2‐dependent mechanisms. Hence, targeting CXCR2 in NKX2‐1‐low tumors is a potential antitumor therapy that may improve LUAD patient outcomes.

## Graphical abstract

An overview showing the utilization of unconventional molecular profiling techniques, such as Single‐cell RNA sequencing (scRNA‐seq) and Visium In situ Capturing, employed to unravel the modulatory role of NK2 homeobox 1 (NKX2‐1) within the immune microenvironment of lung adenocarcinoma (LUAD). The downregulation of NKX2‐1 triggers the expression and secretion of CXCL1, CXCL2, CXCL3, and CXCL5. This increases neutrophil recruitment and infiltration into LUAD tumors, thereby promoting cancer progression.

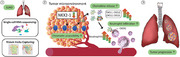

## 1 Introduction

Lung cancer is the most prevalent and aggressive cancer worldwide, with non‐small‐cell lung carcinoma (NSCLC) comprising ≈85% of all cases. In turn, NSCLC can be classified into lung adenocarcinoma (LUAD), squamous cell carcinoma (SCC), and large cell lung carcinoma (LCLC).^[^ ^1^ ^]^ LUAD is a common NSCLC subtype associated with the highest mortality, increased recurrence rate, and short relapse‐free survival.^[^ ^2^ ^]^ Despite the advancements in lung cancer treatment,^[^ ^3^ ^]^ the prognosis of LUAD patients remains poor due to the challenges posed by metastasis and drug resistance.^[^ ^4^ ^]^ High‐grade LUAD is characterized by elevated tumor plasticity, leading to cellular and molecular changes, such as epithelial‐mesenchymal transition (EMT), a process critical for metastasis.^[^ ^5^ ^]^ Previous studies have shown that the alteration of the tumor microenvironment (TME) affects the infiltration of immune cells with concurrent LUAD tumor progression.^[^ ^6^ ^]^ However, the detailed mechanisms governing the LUAD‐associated immune microenvironment, which involves a complex interplay of immune cells, cytokines, and paracrine‐driven molecules interacting with the tumor, remain unclear.

The immune microenvironment comprises an intricate network of immune cells, cytokines, and various molecules interacting with cancer cells within the TME.^[^ ^7^ ^]^ Among them are neutrophils, a type of innate immune cells that play a vital role in responding to infection and inflammation.^[^ ^8^ ^]^ Neutrophils are abundant in the bloodstream, comprising ≈70% of all human white blood cells and 10% – 20% in mice.^[^ ^9^ ^]^ The activation of CXC Motif Chemokine Receptor 2 (CXCR2) is a critical step in triggering the migration of neutrophils from the bone marrow,^[^ ^10^ ^]^ whereas CXCR4 regulates the retention of neutrophils in the bone marrow.^[^ ^11^ ^]^ In solid tumors, neutrophils can exhibit anti‐tumorigenic activity involving cytotoxicity against tumor cells and promote tumor growth, angiogenesis, metastasis, and immune evasion.^[^ ^9^, ^12^ ^]^ The infiltration of tumors with neutrophils is mediated through the chemotactic effects of CXC chemokines by binding to their receptor, CXCR2.^[^ ^13^ ^]^ For instance, CXCL8 is a common ligand of CXCR2 that is mainly secreted by different types of tumors and controls neutrophil recruitment.^[^ ^14^ ^]^ Additionally, the activation of CXCLs/CXCR2 signaling promotes tumor angiogenesis, metastasis, chemoresistance, and tumor progression.^[^ ^15^, ^16^ ^]^

Cell plasticity facilitates the transition between cell lineages and the emergence of drug resistance in specific cancer subtypes, contributing to increased tumor aggressiveness.^[^ ^17^ ^]^ These transitions are characterized by massive changes in gene expression programs regulated by master regulator transcription factors (TFs). Among them is NK2 homeobox 1 (NKX2‐1), which is predominantly expressed during lung and thyroid development^[^ ^18^ ^]^ and serves as a lineage‐specific TF that determines alveolar cell identity.^[^ ^19^ ^]^ In lung cancer, NKX2‐1 regulates the identity of LUAD by enforcing differentiation programs; meanwhile, NKX2‐1 downregulation confers worse disease outcomes in poorly differentiated tumors.^[^ ^20^ ^]^ On the contrary, transdifferentiation from adenomatous to more aggressive squamous histological type is associated with elevated transcriptional activity of SOX2, a well‐known TF in SCC.^[^ ^21^ ^]^ SOX2 is known to change SCC phenotype to a club and alveolar type 2 (AT2) cells.^[^ ^21^ ^]^ Furthermore, it enhances the generation of neural progenitor cells in lung epithelial cells.^[^ ^22^ ^]^ The balance between SOX2 and NKX2‐1 plays a critical role in determining the shifts in cellular lineage that modulate lung cancer progression. This is because lineage‐specific TFs govern differentiation status, which is typically associated with the degree of tumor malignancy.^[^ ^19^ ^]^

Recently, it has been demonstrated that neutrophil plasticity and heterogeneity underlie adverse events that may result in the discontinuation of immunotherapy.^[^ ^9^ ^]^ Previous reports have shown that SOX2 is a critical TF that recruits neutrophils into the TME by regulating CXCL3 and CXCL5 expression, thus facilitating cancer progression.^[^ ^17^ ^]^ However, the implication of NKX2‐1 in attracting immune cells to the TME and mediating the progression of LUAD remains unclear. Here, we demonstrated that low expression of NKX2‐1 correlates with high neutrophil infiltration, which predicts poor clinical outcomes in LUAD patients. Our single‐cell and Visium in situ capturing NGS analyses revealed a strong cell‐cell communication between NKX2‐1‐low tumors and neutrophils through the activation of CXCLs/CXCR2 signaling. The inhibition of CXCR2 chemokine receptor with specific inhibitor SB225002 decreased the infiltration of tumor‐promoting neutrophils, resulting in reduced tumor growth in NKX2‐1‐low tumors.

## 2 Results

### 2.1 Low Expression of NKX2‐1 is Associated with Aggressive LUAD

**Figure 1:**
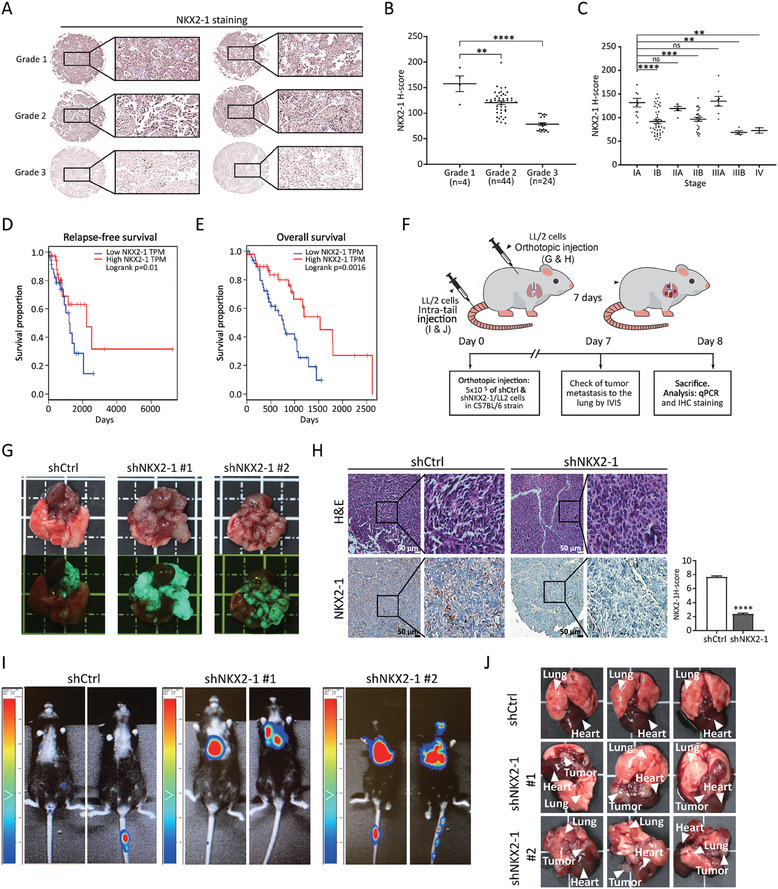
Low expression of NKX2‐1 is associated with aggressive LUAD. A) A representative image of NKX2‐1 IHC staining performed on tumor tissue microarrays of different grades of LUAD. B and C) Histoscore (H‐score) quantification of NKX2‐1 IHC staining performed on a tissue microarray of different LUAD grades (B) and stages (C). The data are presented as means ± SD error bars, *N* = 3, **p < 0.01, ***p < 0.001, ****p < 0.0001, ns – not significant (Student's t‐test). D and E) Kaplan‐Meier curves showing the overall survival (D) and disease‐free survival of NKX2‐1 LUAD patients from the TCGA dataset (E). *p*‐values were calculated by the log‐rank test. F) Schematic representation showing the design of experiments performed on both orthotopic and tail vein injected mouse models by using shNKX2‐1/LL2 and shCtrl/LL2 cells. G) Photographs (top panels) and GFP fluorescence images (bottom panels) of tumors derived from orthotopically injected shCtrl/LL2 (left panel) and shNKX2‐1/LL2 cells (middle and right panels). H) Hematoxylin‐eosin staining and IHC staining of NKX2‐1 were performed on the tumors derived from orthotopically injected shCtrl/LL2 and shNKX2‐1/LL2 cells. Left panel: 2197 representative images. Right panel: H‐score quantification. The data are presented as means with SD error bars, *N* = 3, ****p < 0.0001 (Student's t‐test). I and J) Bioluminescent signal visualization (I) and photographs (J) of tumors derived from shCtrl/LL2 and shNKX2‐1/LL2 cells injected via the tail vein.

Downregulation of NKX2‐1 is known to be associated with poorly differentiated lung adenocarcinoma (LUAD) tumors exhibiting high metastatic potential. Conversely, NKX2‐1 is upregulated in less malignant, well‐differentiated LUAD tumors.^[^ ^23^ ^]^ To validate the effects of NKX2‐1, we investigated the protein expression levels of NKX2‐1 across different grades of LUAD by conducting immunohistochemistry (IHC) staining on LUAD tissue microarray. As can be seen on the representative IHC images (**Figure** **1**A) or from quantification of the IHC staining (Figure 1B), the protein expression of NKX2‐1 in grade 3 LUAD tissues is markedly lower compared to grade 1 and 2 tissues. Moreover, quantification of IHC staining based on LUAD staging showed that stage IIIB and stage IV LUAD tissues were characterized by lower expression of NKX2‐1 compared to stage IA, IB, IIA, IIB, and IIIA LUAD tissues (Figure 1C). Furthermore, the survival analysis using The Cancer Genome Atlas (TCGA) dataset showed that high NKX2‐1 expression levels correlated with better relapse‐free survival and overall survival outcome in LUAD (Figure 1D,E).

In this study, we sought to explore the regulatory role of NKX2‐1 in vivo tumorigenicity by using both the orthotopic LUAD mouse model and the experimental model of LUAD distal metastasis. NKX2‐1 was knocked down in murine Lewis lung carcinoma cells (LL2) expressing luciferase reporter and eGFP. qRT‐PCR showed the knockdown of NKX2‐1 in LL2 (shNKX2‐1/LL2) cells with 60–70% efficiency compared to scrambled control shRNA (shCtrl/LL2) cells (Figure S1A, Supporting Information). These cells were subjected either to orthotopic implantation to the lung or intra‐tail injection (Figure 1F). The orthotopically implanted shNKX2‐1/LL2 cells generated tumors characterized by increased growth in the recipient mice compared to the shCtrl/LL2 implanted cells (Figure 1G). IHC staining of NKX2‐1 was carried out in the excised tumor tissues to validate the expression level of NKX2‐1. The result showed low levels of NKX2‐1 in shNKX2‐1/LL2‐derived tumor cells when compared with the shCtrl/LL2‐derived tumor cells (Figure 1H). Additionally, immunoblotting and qRT‐PCR were also performed to validate the expression of NKX2‐1 in the excised tumor tissues (Figure S1B,C, Supporting Information). To validate prior studies that showed the regulatory role of NKX2‐1 in epithelial‐mesenchymal transition (EMT),^[^ ^5^ ^]^ IHC staining of some EMT markers was performed on the excised tumor tissues. The results showed the upregulation of fibronectin and the downregulation of E‐cadherin in the tumors derived from shNKX2‐1/LL2 cells when compared to shCtrl/LL2 cells. This indicated the induction of EMT after NKX2‐1 knockdown (Figure S1D, Supporting Information).

To demonstrate the metastatic potential in the orthotopic model, we intravenously injected shCtrl/LL2 or shNKX2‐1/LL2 cells into the mice via the tail vein (Figure 1F). The mice injected with shNKX2‐1/LL2 cells exhibited a significant increase of luciferase signal at the upper torso compared to the shCtrl/LL2‐injected mice (Figure 1I). This result was in line with the considerable tumor nodules observed in the lungs of shNKX2‐1/LL2 but not shCtrl/LL2‐injected mice (Figure 1J). These results indicated that low expression of NKX2‐1 in LUAD contributed to increased tumor growth (Figure 1G) and higher metastasis potential (Figure 1I). In summary, the clinical data, cellular models, and animal experiments demonstrated that low expression of NKX2‐1 was associated with features related to advanced cancer status and worse clinical outcomes.

### 2.2 NKX2‐1 Expression Negatively Correlates with Neutrophil Infiltration

**Figure 2:**
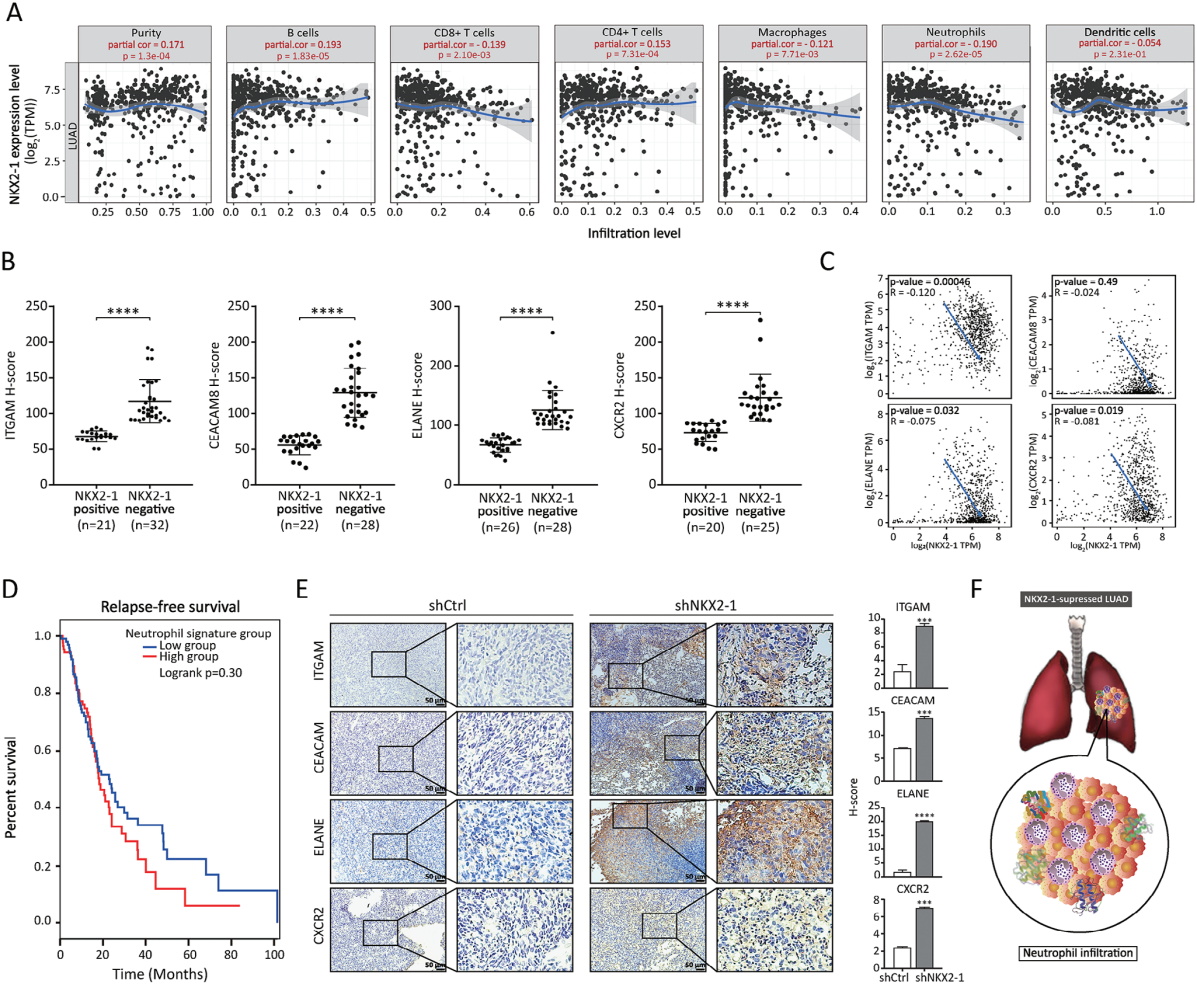
NKX2‐1 expression negatively correlates with neutrophil infiltration. A) Scatter plots showing the correlation between NKX2‐1 expression and the indicated immune cell infiltrate levels in LUAD from TIMER dataset analysis. The gene expression levels against tumor purity are displayed first because genes highly expressed in the TME are expected to exhibit negative associations with tumor purity and vice versa. B) H‐score quantification of the expression levels of the selected neutrophil markers (ITGAM, CEACAM8, ELANE, and CXCR2) performed on tumor tissue microarrays of NKX2‐1‐positive and negative LUAD tissues. The data are presented as means with SD error bars, ****p < 0.0001 (Student's t‐test). C) The TCGA dataset analysis shows the correlation between NKX2‐1 and the selected neutrophil markers (*ITGAM*, *CEACAM8*, *ELANE*, and *CXCR2*) in LUAD. D) Relapse‐free survival curve showing the survival of LUAD patients with high or low neutrophil infiltration from the TCGA dataset analysis. *p*‐values were calculated by the log‐rank test. E) IHC staining of the indicated neutrophil markers in the cross sections of shCtrl/LL2 and shNKX2‐1/LL2‐derived tumors from mouse orthotopic model. Left panel: representative images. Right panel: H‐score quantification. The data are presented as means with SD error bars, *N* = 3, ***p < 0.001, ****p < 0.0001 (Student's t‐test). F) A schematic illustration showing that NKX2‐1‐low LUAD tumors are characterized by high infiltration of neutrophils.

The tumor microenvironment (TME) can affect clinical outcomes due to the ability of innate or adaptive immune cells to exert either tumor‐promoting or tumor‐suppressing effects.^[^ ^24^ ^]^ The most abundant immune cells that infiltrate tumors are neutrophils and macrophages, which may exert pro‐tumorigenic effects by driving angiogenesis, extracellular matrix remodeling, metastasis, and immunosuppression.^[^ ^25^ ^]^ Therefore, we aimed to elucidate the impact of NKX2‐1 on the complex interactions between malignant and immune cells within the TME. Tumor Immune Estimation Resource (TIMER) dataset^[^ ^26^ ^]^ was used to systematically analyze the correlation between NKX2‐1 differential expression and immune cell infiltrations. The analysis showed that the infiltration of CD8^+^ T cells, macrophages, neutrophils, and dendritic cells negatively correlated with NKX2‐1 expression levels in LUAD (**Figure** **2**A). Given that the correlation with neutrophil infiltration was the most statistically significant and given the growing evidence of its oncogenic role in LUAD,^[^ ^8^, ^18^, ^23^ ^]^ we analyzed the expression of some neutrophil markers such as ITGAM, CEACAM8, ELANE, and CXCR2 by performing an IHC staining in NKX2‐1‐negative and positive LUAD tissues. The representative images (Figure S2A, Supporting Information) and the quantification of the IHC staining (Figure 2B) showed an upregulation of these markers in NKX2‐1‐negative LUAD tissues compared to NKX2‐1‐positive tissues. Furthermore, the TCGA dataset analysis confirmed the negative correlation of NKX2‐1 expression with the selected neutrophil markers (ITGAM, CEACAM8, ELANE, and CXCR2) in LUAD patients (Figure 2C). In addition, the survival analysis showed that low infiltration of neutrophils in relapse‐free LUAD patient tumors was associated with better survival outcomes (Figure 2D). Moreover, IHC staining of the tumor tissues derived from shNKX2‐1/LL2 cells orthotopically implanted into mouse lungs (Figure 1G) also showed elevated expression of ITGAM, CEACAM8, ELANE, and CXCR2 as compared to the control (Figure 2E). In conclusion, our data suggest that low expression of NKX2‐1 correlates with high neutrophil infiltration and the abundance of neutrophil infiltration predicts poor clinical outcomes for LUAD patients (Figure 2F).

### 2.3 NKX2‐1‐Low Tumors Promote Neutrophil Infiltration via CXC Chemokines

**Figure 3:**
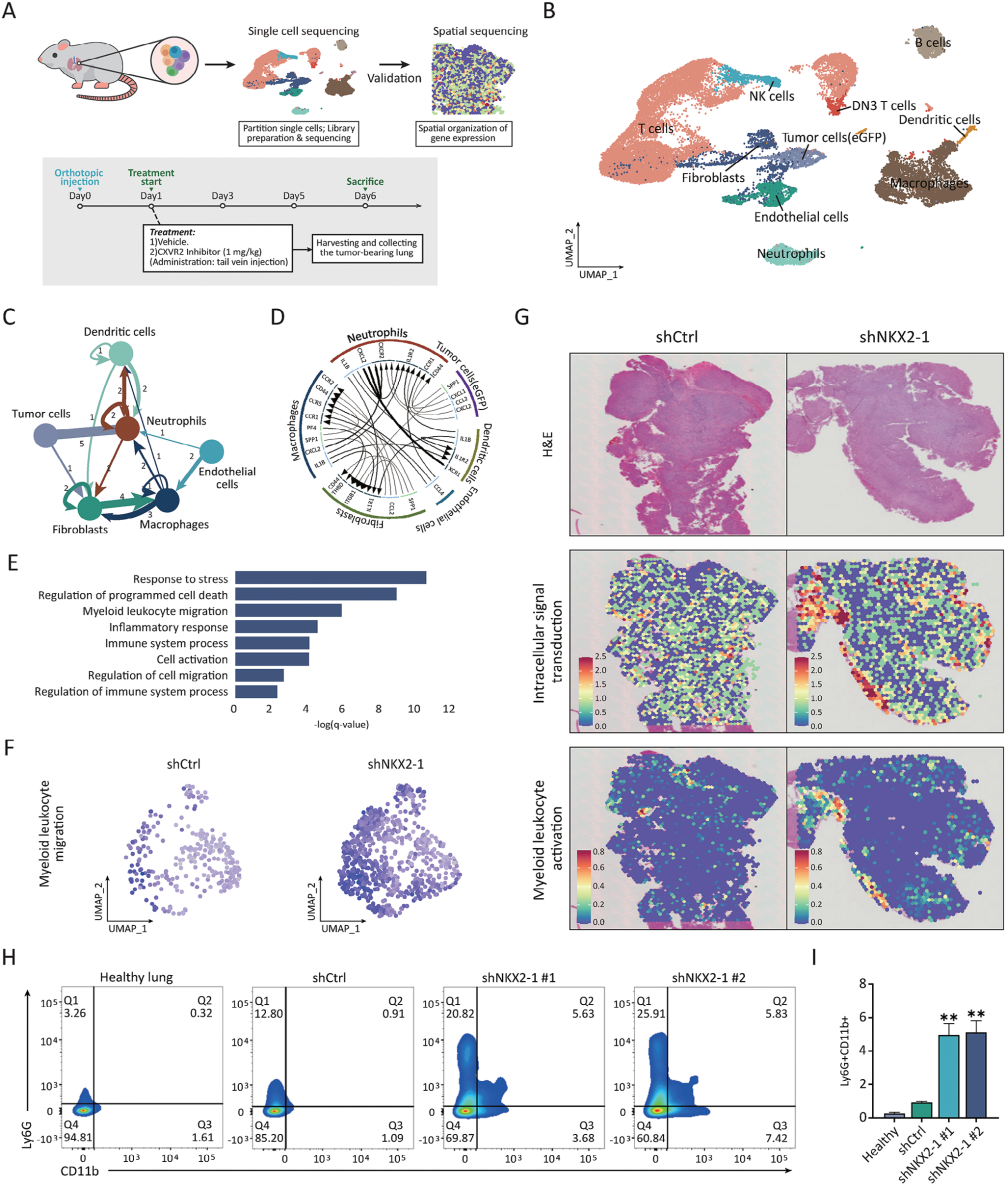
NKX2‐1‐low tumors promote neutrophil infiltration via CXC chemokines. A) Diagram demonstrating the experimental procedure for scRNA‐seq and Visium in situ capturing. B) UMAP analysis from scRNA‐seq showing the distinct cell populations across 2 samples (shCtrl/LL2 and shNKX2‐1/LL2‐derived tumors). C and D) iTALK analysis of scRNA‐seq validating the crosstalk between tumor cells and immune cells (C) and cell‐to‐cell communication (D) between cell types and the involved genes. E) Gene ontology biological process (GO‐BP) enrichment analysis of differentially expressed genes in shNKX2‐1/LL2 tumors compared to shCtrl/LL2 tumors identified by scRNA‐seq. F) UMAP analysis from scRNA‐seq showing myeloid leukocyte migration in shCtrl/LL2 and shNKX2‐1/LL2 tumors. G) Hematoxylin‐eosin staining (H&E) section annotations in shCtrl/LL2 and shNKX2‐1/LL2 tumors (top panel) and representative image from Visium in situ capturing showing the activation of intracellular signal transduction and myeloid leukocyte activation. (H and I) Flow cytometry identification of Ly‐6G^+^CD11b^+^ cells from healthy lungs and metastasized tumor‐bearing lungs (shCtrl/LL2 and shNKX2‐1/LL2) H). The quantification of Ly‐6G^+^CD11b^+^ cells (I) is shown as means with SD error bars, **p < 0.01 (Student's t‐test).

Chemokines secreted by both tumor cells and the TME play a central role in regulating the recruitment of immune cells such as neutrophils.^[^ ^27^ ^]^ Notably, the infiltration of tumor‐promoting neutrophils leads to increased tumor progression^[^ ^27^ ^]^ and is generally associated with high‐grade aggressive tumors.^[^ ^28^ ^]^ Given that this observation is not always the same in all tumors due to their striking heterogeneity, we performed single‐cell RNA sequencing (scRNA‐seq) and Visium in situ capturing profiling on shCtrl/LL2 and shNKX2‐1/LL2‐derived tumor tissues to comprehensively characterize the infiltrated immune cells upon NKX2‐1 knockdown at high resolution. For scRNA‐seq, shCtrl/LL2 and shNKX2‐1/LL2 cells were orthotopically implanted into mouse lungs, and the harvested tumors were dissociated into viable single cells (**Figure** **3**A), which were further shown to pass standard scRNA‐seq quality control (Figure S3A–C, Supporting Information).

To further characterize the distinct cell types from shCtrl/LL2 and shNKX2‐1/LL2‐derived tumors, preliminary clustering was applied to all the cells by using uniform manifold approximation and projection (UMAP) analysis, and the FindAllMarkers package was used to find the different markers in each cluster (Figure 3B). Meanwhile, the crosstalk between tumor cells and various cell types of the TME was further analyzed by using iTALK R package (Figure 3C,D). The results indicated a significantly strong interaction of tumor cells with neutrophils (Figure 3C). Further analysis of the cell‐cell interactions indicated communication between tumor cells and neutrophils through CXCLs/CXCR2 signaling activation (Figure 3D). The enrichment analysis of gene ontology biological processes (GO‐BP) showed the enrichment in such terms as a response to stress, regulation of programmed cell death, myeloid leukocyte migration, and inflammatory response (Figure 3E). UMAP analysis revealed the increase of myeloid leukocyte migration in NKX2‐1‐knocked down cells from our scRNA‐seq as indicated from enriched GO term (Figure 3F), indicative of the increased myeloid cells migration to shNKX2‐1/LL2 tumors when compared to shCtrl/LL2 tumors. To further validate the infiltration of immune cells in NKX2‐1‐low tumors, we performed a spatially resolved transcriptomics analysis^[^ ^29^ ^]^ with Visium in situ capturing profiling of shCtrl/LL2 and shNKX2‐1/LL2 tumors. The tissues were collected with a capture area of 8 × 8 mm and ≈5000 gene expression spots were chosen (Figure 3G). The results also showed an increase in myeloid leukocyte activation and intracellular signal transduction upon NKX2‐1 downregulation (Figure 3G). To validate the infiltration of neutrophils, we applied flow cytometry analysis to identify CD11b+ Ly‐6G+ cells, commonly recognized as tumor‐associated neutrophils (TANs).^[^ ^33^, ^34^ ^]^ We observed a significant increase in CD11b+ Ly‐6G+ neutrophils in tumor‐bearing lungs from the recipient mice intravenously injected with either clone of shNKX2‐1/LL2 cells, compared to control mice transplanted with shCtrl/LL2 cells or the healthy lungs (Figure 3H,I). Altogether, our findings demonstrated an increase in neutrophil infiltration within NKX2‐1‐low tumors, potentially mediated by the activation of cell surface receptor signaling pathways.

### 2.4 NKX2‐1 Downregulation Affects the Expression of CXC Chemokines

**Figure 4:**
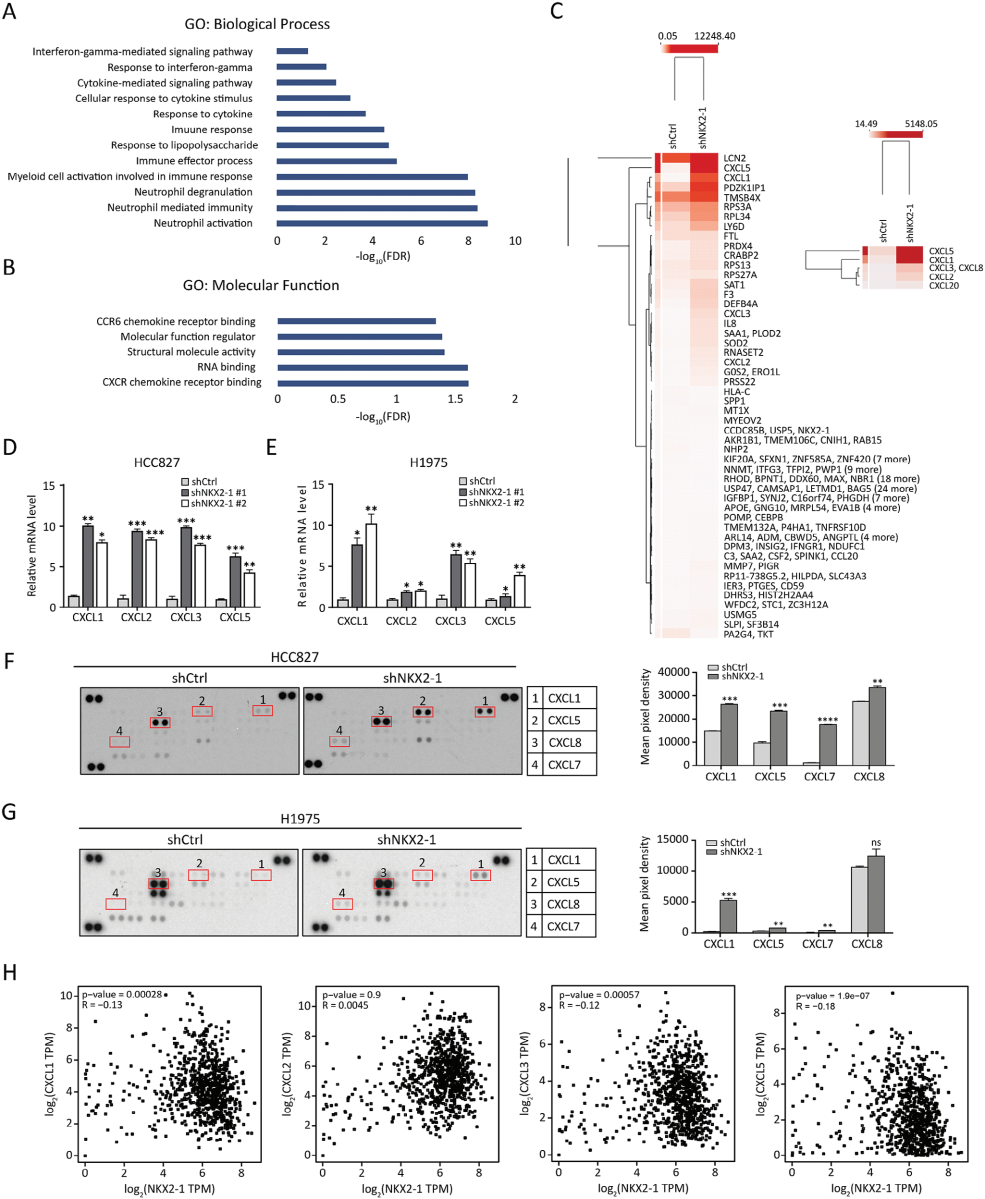
NKX2‐1 downregulation affects the expression of CXC chemokines. A and B) Functional enrichment analysis showing the enrichment of GO biological process (GO‐BP) (A) and molecular function (GO‐MF) (B) terms among the genes positively regulated by NKX2‐1 knockdown. C) Hierarchical clustering heatmap showing the differential gene expression (left panel) and the expression of CXC chemokines (right panel) upon NKX2‐1 knockdown. D and E) qRT‐PCR analysis showing the expression levels of CXC chemokines in HCC827 (D) and H1975 (E) cells subjected to NKX2‐1 knockdown. The mean fold changes (N = 3) relative to scrambled shRNA control (shCtrl) are shown with SD error bars, *p < 0.05, **p < 0.01, ***p < 0.001 (Student's t‐test). F and G) Chemokine array analysis showing the secretion levels of the indicated chemokines following NKX2‐1 knockdown in HCC827 (F) and H1975 cells (G). Left panel: representative dot plot. Right panel: densitometry quantification of dot blots. *p < 0.05, **p < 0.01, ***p < 0.001, ns – not significant (Student's t‐test). H) The TCGA dataset analysis shows the correlation between NKX2‐1 expression and *CXCL1*, *CXCL2*, *CXCL3*, and *CXCL5* mRNA expression levels.

Having illustrated the role of CXC chemokines in mediating cell‐cell communication between tumor cells and neutrophils by scRNA‐seq analysis (Figure 3E), we proceeded to assess the regulatory role of NKX2‐1 on CXC chemokine expression and secretion in human LUAD cell culture model. We efficiently knocked down NKX2‐1 expression in NKX2‐1‐high LUAD cell lines (HCC827 and H1975) using shRNA constructs (Figure S2B,C, Supporting Information). RNA‐seq analysis was performed on HCC827 cells subjected to shRNA‐mediated NKX2‐1 knockdown. Functional enrichment analysis of the genes positively regulated by NKX2‐1 knockdown revealed neutrophil activation, neutrophil‐mediated immunity, and myeloid cell activation involved in immune response as the most enriched GO‐BP terms (**Figure** **4**A). Meanwhile, the most enriched GO molecular function (GO‐MF) terms were related to CXCR chemokine receptor binding, and CCR6 chemokine receptor binding (Figure 4B). These cell culture model observations on the role of CXC chemokines in neutrophil recruitment were consistent with our in vivo mouse model upon the functional elimination of NKX2‐1 (Figure 3D). More specifically, our RNA‐seq analysis identified the upregulation of several neutrophil chemotactic genes such as *CXCL1*, *CXCL2*, *CXCL3*, *CXCL5*, and *CXCL8* upon NKX2‐1 knockdown (Figure 4C). Among them, CXCL8 has been extensively characterized as a mediator of neutrophil mobilization and attraction^[^ ^30^ ^]^; therefore, we decided to explore the role of other CXC chemokines in NKX2‐1‐low tumors. qRT‐PCR analysis was carried out to validate the RNA‐seq data, and it confirmed that the knockdown of NKX2‐1 by 2 shRNAs contributed to the upregulation of *CXCL1*, *CXCL2*, *CXCL3*, and *CXCL5* mRNA levels in HCC827 and H1975 LUAD cells (Figure 4D,E). Furthermore, the chemokine array showed that the knockdown of NKX2‐1 in HCC827 and H1975 cells resulted in increased secretion of CXCL1, CXCL5, CXCL7, and CXCL8 (Figure 4F,G). However, there was no significant increase in CXCL8 secretion in NKX2‐1/H1975 cells as compared to shCtrl/H1975 cells (Figure 4G). The TCGA dataset analysis also indicated a negative correlation between the expression of NKX2‐1 and *CXCL1*, *CXCL3*, and *CXCL5*. However, no correlation was observed between the expression of *CXCL2* and NKX2‐1 (Figure 4H). To summarize, our data demonstrated the role of NKX2‐1 as a suppressor of CXC chemokine expressions and secretions in LUAD cells.

### 2.5 NKX2‐1 Suppresses the Chromatin Accessibility at the Promoter Regions of CXC Chemokine Genes

**Figure 5:**
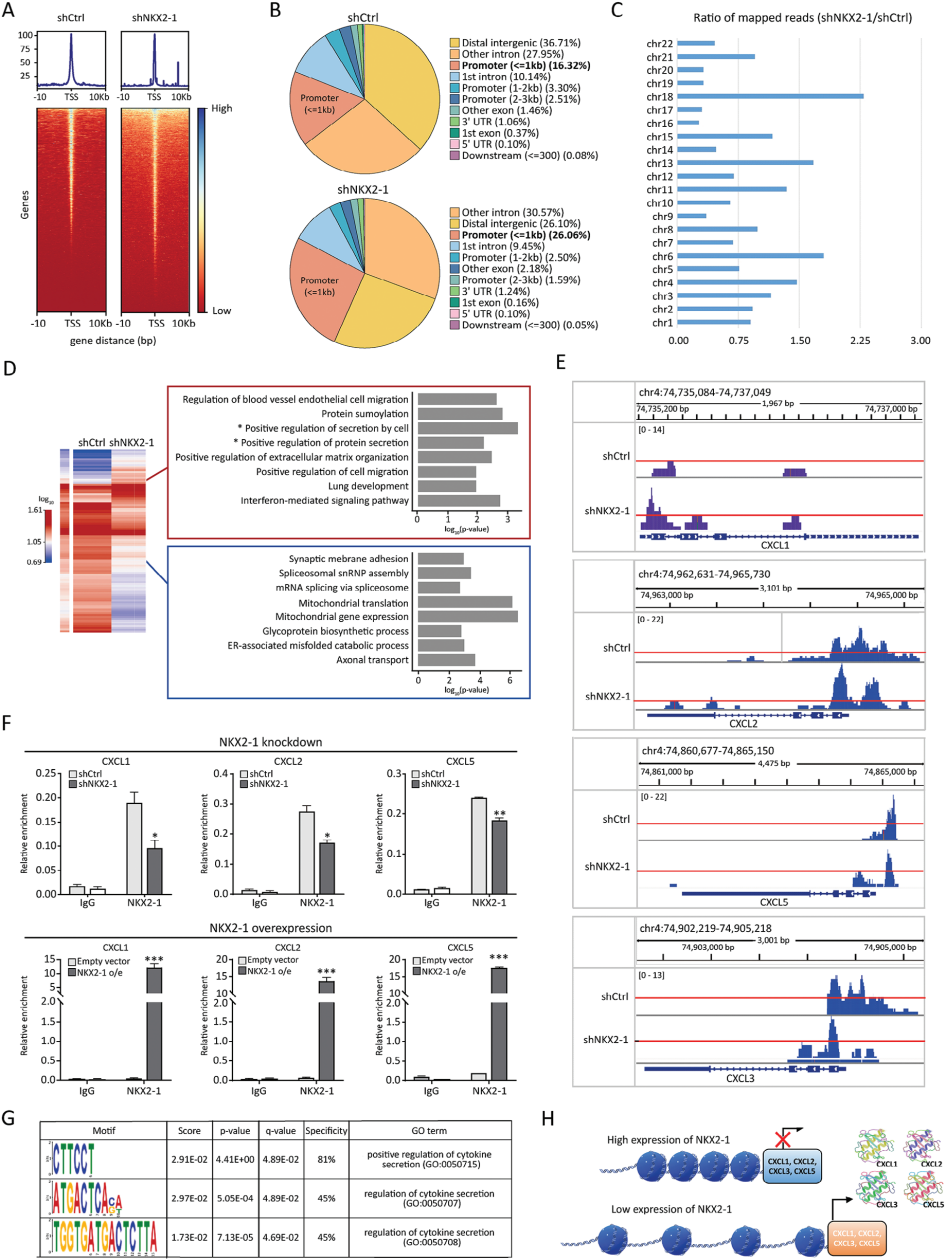
NKX2‐1 suppresses the chromatin accessibility at the promoter regions of CXC chemokine genes. A) Heatmap representation of the chromatin accessibility at multiple genes' transcription start sites (TSS) and 10 kb upstream and downstream regions. B) Annotation for ATAC‐seq peak localization showing the distribution of sequenced reads across the indicated functional genomic elements. The peaks located 1 Kb upstream and 100 bp downstream of the TSS were defined as promoter‐TSS. C) The distribution of open chromatin areas across the chromosomes is shown as the ratio of signals from shNKX2‐1/H1975 versus shCtrl/H1975. D) GO‐BP analysis of genes with open (top panel) and closed chromatin structure (bottom panel) at the promoter regions resulting from NKX2‐1 knockdown. E) Integrative Genomics Viewer (IGV) representation of ATAC‐seq signals at the promoter regions of CXC chemokine genes in shCtrl/H1975 and shNKX2‐1/H1975 samples. The red line denotes a given range across the 2 samples. F) ChIP‐qPCR analysis showing the binding of NKX2‐1 at the promoter regions of *CXCL1*, *CXCL2*, and *CXCL5* genes in shNKX2‐1/H1975 cells and H1975/AZDR overexpressing NKX2‐1. Mean fold changes (*N* = 3) relative to input are shown with SD error bars *p < 0.05, **p < 0.01, ***p < 0.001 (Student's t‐test). G) The most enriched canonical NKX2‐1 binding motifs across ATAC‐seq reads. H) Schematic illustration showing the potential mechanism of NKX2‐1 regulating the expression of CXC chemokines by altering chromatin structure.

Following the observations that NKX2‐1 suppresses the expression of various CXC chemokines in LUAD cells (Figure 4), we sought to elucidate the mechanism involved in NKX2‐1‐mediated suppression of CXC chemokine expression. NKX2‐1 is a homeobox transcription factor that activates the transcription of thyroid and lung‐specific genes.^[^ ^31^ ^]^ Through immunofluorescence staining, we confirmed the predominant nuclear localization of NKX2‐1 in HCC827 and H1975 cells (Figure S2D, Supporting Information). Thus, we hypothesized that NKX2‐1 could exert its action by affecting the chromatin structure. The open chromatin regions in shCtrl/H1975 and shNKX2‐1/H1975 cells were sequenced by an assay for transposase‐accessible chromatin using sequencing (ATAC‐seq), a sequencing method based on the insertion of sequencing adapters by hyperactive Tn5 transposase.^[^ ^32^ ^]^ To evaluate the effect of NKX2‐1 on the overall pattern of chromatin accessibility, we estimated the abundance of reads with increased and decreased accessibility in shCtrl/H1975 and shNKX2‐1/H1975 samples. Indeed, the open chromatin ATAC‐seq reads tended to be enriched with a large number of genes at the transcription start sites (TSS) in shNKX2‐1/H1975 samples in comparison with shCtrl/H1975, indicative of the potential role of NKX2‐1 as a negative gene regulator (**Figure** **5**A).

Generally, the distribution pattern of open chromatin peaks across different functional genomic elements exhibited an increase within less than 1 kb of the promoter regions in shNKX2‐1 sample (26.06%) when compared to the shCtrl/H1975 (16.32%) (Figure 5B). To determine the changes in chromatin accessibility following NKX2‐1 knockdown, we evaluated the differential accessibility between shNKX2‐1/H1975 and shCtrl/H1975 cells across the genome (Figure 5C). The enriched open chromatin regions resulting from NKX2‐1 knockdown were predominantly located on chr18, chr13, chr6, and chr4 (Figure 5C). Interestingly, chr4, where the cluster of CXC chemokine genes is located, was one of the most chromatin‐accessible chromosomes resultant in NKX2‐1 knockdown. GO‐BP enrichment analysis revealed that the enriched open chromatin regions resulting from NKX2‐1 knockdown were mostly located at the promoter regions of genes involved in the positive regulation of secretion by cells and positive regulation of protein secretion (Figure 5D). On the other hand, more closed chromatin structure in shNKX2‐1/H1975 cells as compared to shCtrl/H1975 cells was illustrated at the promoter regions of the genes associated with mitochondrial gene expression and mitochondrial translation (Figure 5D).

Notably, our ATAC‐seq results showed an increase in open chromatin‐associated reads at the promoter regions of *CXCL1*, *CXCL2*, and *CXCL5* genes following the knockdown of NKX2‐1 in H1975 cells (Figure 5E). This suggested that NKX2‐1 could potentially modulate the chromatin structure at the promoter regions of these chemokine genes. We validated this observation using ChIP‐qPCR, which demonstrated that NKX2‐1 knockdown in H1975 (NKX2‐1‐high cell line) resulted in reduced NKX2‐1 occupancy at the promoter regions of *CXCL1*, *CXCL2*, and *CXCL5* (Figure 5F). In contrast, the overexpression of NKX2‐1 in H1975/AZDR cells (NKX2‐1‐low cell line) increased NKX2‐1 occupancy at the promoter regions of the same genes (Figure 5F). Next, we sought to identify the potential NKX2‐1‐binding sequence motifs that might be related to the regulation of cytokines expression. ATAC‐seq reads were analyzed by using Find Individual Motif Occurrences (FIMO) software to predict NKX2‐1‐binding motif sequences. Our results showed the 3 most prevalent NKX2‐1‐binding motif sequences in ATAC‐seq read close to the canonical motif sequence (Figure 5G). Collectively, our data suggest that the direct binding of NKX2‐1 negatively regulates chromatin accessibility at the promoters of CXC chemokine genes (Figure 5H), which is consistent with the regulatory role of NKX2‐1 on CXC chemokine expression and secretion (Figure 4).

### 2.6 Neutrophils Attracted by NKX2‐1‐Low Tumors Exhibit Cancer‐Promoting Properties

**Figure 6:**
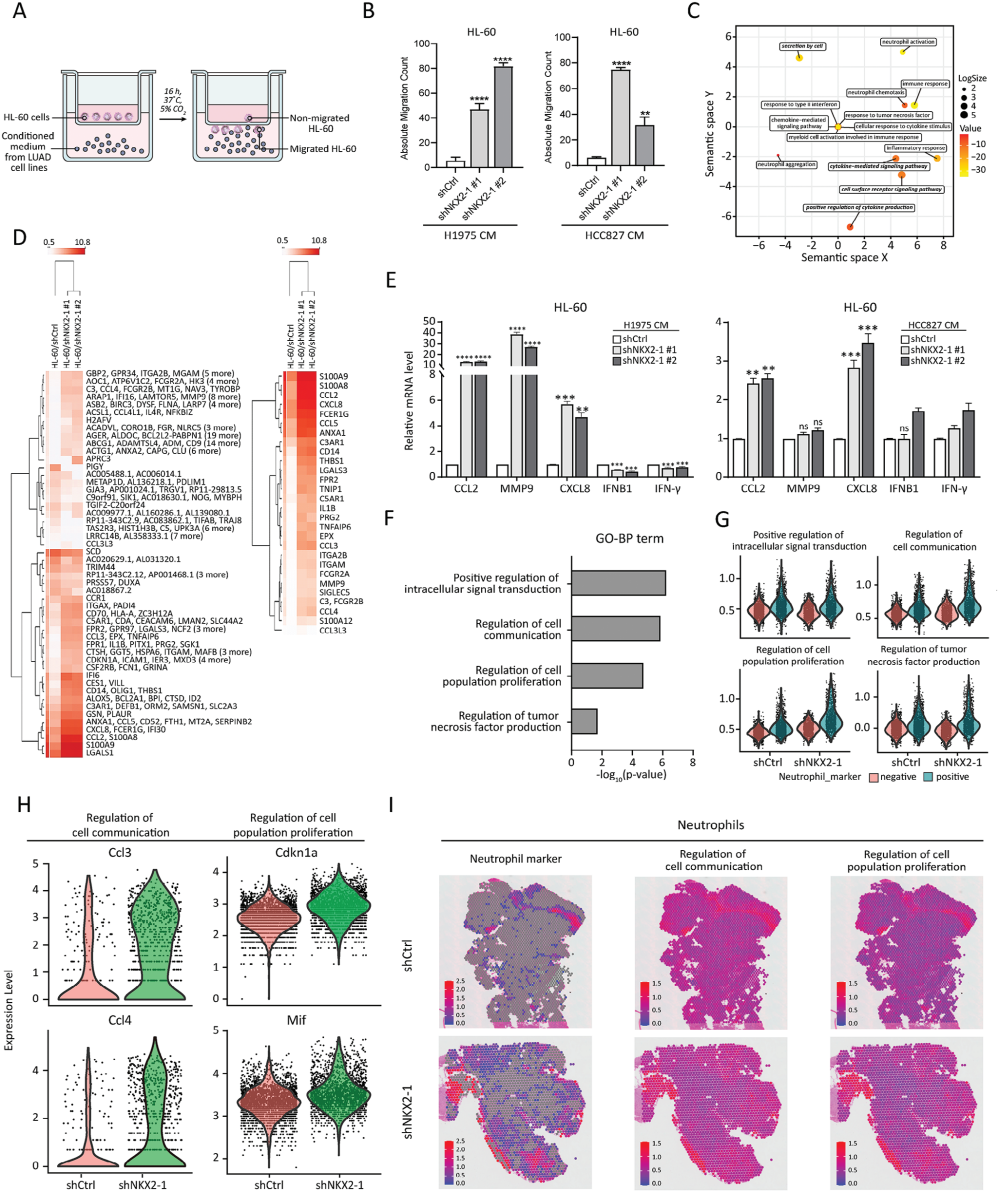
Neutrophils attracted by NKX2‐1‐low tumors exhibit cancer‐promoting properties. A) The schematic illustration demonstrating the experimental procedure for chemotaxis assay using HL‐60 and conditioned medium from LUAD cell lines. B) Chemotaxis assay showing the migratory capacity of HL‐60 cells in response to the conditioned media (CM) derived from H1975 (left panel) and HCC827 (right panel) subjected to NKX2‐1 knockdown. Mean numbers of migrated cells (N = 3) are shown with SD error bars, *p < 0.05, **p < 0.01, ***p < 0.001 (Student's t‐test). C) Revigo scatter plot showing enriched GO‐BP terms among the genes upregulated in HL‐60 cells cultured with the conditioned media derived from shNKX2‐1/H1975 as compared to shCtrl/H1975 as shown by RNA‐seq analysis. D) Hierarchical clustering heatmap from RNA‐seq showing the differential gene expression (left panel) and neutrophil‐related genes (right panel) in HL‐60 cultured with the conditioned media derived from shCtrl/H1975 and shNKX2‐1/H1975 cells. E) qRT‐PCR analysis showing the expression levels of pro‐inflammatory genes and anti‐inflammatory genes in HL‐60 cultured with the conditioned media derived from shCtrl/H1975 and shNKX2‐1/H1975 cells. The mean fold changes (N = 3) relative to shCtrl are shown with SD error bars, **p < 0.01, ***p < 0.001, ****p < 0.0001, ns – not significant (Student's t‐test). F) GO‐BP enrichment analysis of the genes upregulated in the tumor regions derived from shNKX2‐1/LL2 cells compared to shCtrl/LL2 cells from Visium in situ capturing profiling. G) GO‐BP enrichment analysis of the upregulated genes in tumors derived from shNKX2‐1/LL2 cells compared to shCtrl/LL2 from Visium in situ capturing profiling. The genes upregulated with ‐log2 (fold‐change) of more than 1 were defined as infiltration‐positive, while the genes with below‐threshold expression were defined as infiltration‐negative. H) Violin plots showing the expression levels of the most upregulated genes annotated by GO‐BP terms “regulation of cell communication” and “regulation of cell population proliferation” in shNKX2‐1 and shCtrl infiltrated neutrophils. I) Visium in situ capturing representative images showing the neutrophil‐positive regions and overlapping regions of the expression of genes annotated by “regulation of cell communication” and “regulation of cell population proliferation” GO‐BP terms.

In light of the known role of CXC chemokines as potent neutrophil chemoattractants,^[^ ^33^ ^]^ we conducted a chemotaxis assay to validate the functionality of NKX2‐1‐suppressed CXC chemokines (**Figure** **6**A). As was shown by the transwell chemotaxis assay, HL‐60 cell migration was significantly stimulated by the medium conditioned by shNKX2‐1‐transfected H1975 and HCC827 cells in contrast to the medium conditioned by shCtrl‐transfected cells (Figure 6B). To investigate the detailed global expression programs occurring in the recruited neutrophils, we performed RNA‐seq analysis on HL‐60 cells cultured in the medium conditioned by shCtrl and shNKX2‐1‐transfected H1975 cells. Revigo tool^[^ ^40^ ^]^ was utilized for the GO analysis of the genes positively regulated in HL‐60 cells upon culturing with the conditioned medium from shNKX2‐1/H1975 cells. The results showed the enrichment of GO‐BP terms such as secretion by cells, neutrophil activation, inflammatory response, immune response, neutrophil chemotaxis, etc. (Figure 6C).

The phenotypic properties of neutrophils are associated with their ability to adapt to different inflammatory contexts in the TME. For instance, previous reports have demonstrated that tumor‐promoting or tumor‐suppressing functions of neutrophils are determined by the functional characteristics exhibited through specific markers^[^ ^34^ ^]^ related to neutrophil activation and cytokine status.^[^ ^35^ ^]^ To characterize the neutrophil phenotypes, we applied hierarchical clustering to identify the distinct gene expression patterns in HL‐60 cells cultured with the medium conditioned by NKX2‐1 knockdown cells. The analysis showed an increase in the expression of specific pro‐inflammatory genes such as *CCL3*, *CCL5*, *IL1B*, *CXCL8*, and *CCL4*, among others (Figure 6D). Earlier studies have indicated that the antitumor phenotype (N1) of TANs is marked by elevated levels of *TNFα*, *CCL3*, *ICAM‐1*, and a decreased level of arginase. Conversely, the pro‐tumor phenotype (N2) of TANs is characterized by high levels of *CCL2*, *CCL3*, *CCL4*, *CCL8*, *CCL12*, *CCL17*, *CXCL1*, *CXCL2*, *CXCL8*, and *CXCL16* chemokines.^[^ ^36^ ^]^ To confirm the phenotypic effects on HL‐60 cells cultured in the conditioned medium derived from shNKX2‐1‐transfected H1975 and HCC827 cells, we conducted qRT‐PCR analysis. The results revealed a significant increase in the mRNA expression of *CCL2*, *MMP9*, and *CXCL8* (Figure 6E). However, HL‐60 treated by shNKX2‐1/H1975‐conditioned medium exhibited a significant decrease in the mRNA expression of *IFNβ1* and *IFN‐γ*. In contrast, shNKX2/HCC827 medium showed no significant impact on the expression levels of these genes (Figure 6E).

Furthermore, Visium in situ capturing profiling was performed to identify and characterize the transcriptomes of the infiltrated neutrophils in an in vivo mouse model. The GO‐BP enrichment analysis of the genes overexpressed in tumors derived from shNKX2‐1/LL2 cells as compared to shCtrl/LL2 cells, showed the prevalence of processes such as positive regulation of intracellular signal, regulation of cell communication, regulation of cell population proliferation, and regulation of tumor necrosis factor superfamily cytokine production (Figure 6F). Further analysis of tumor cross‐sections with enriched neutrophil infiltrations revealed the upregulation of neutrophil‐related genes such as *S100a9*, *Serpine1*, *Mt2*, *Nos2*, *Mt1*, *IL33*, *Adm*, and *Ero1l*.^[^ ^37^, ^38^, ^39^ ^]^ The regions with increased above‐threshold expression of these neutrophil‐related genes (‐log2 (fold‐change) > 1) were defined as infiltration‐positive, while the regions with below‐threshold expression were defined as infiltration‐negative. Our results revealed that neutrophil‐positive regions demonstrated the enrichment in cell communication and cell population proliferation GO‐BP terms compared to the neutrophil‐negative regions in both shCtrl and shNKX2‐1 tumors (Figure 6G). Furthermore, the neutrophil‐negative regions exhibited a slight difference based on the GO‐BP terms related to the regulation of cell communication and cell population proliferation (Figure 6G). Our results also revealed that positive regulation of intracellular signal transduction and the regulation of tumor necrosis factor production were not affected in neutrophil‐positive regions (Figure 6G). *Ccl3* and *Ccl4* were the most upregulated genes related to the regulation of cell communication in shNKX2‐1 tumor‐infiltrated neutrophils when compared to the shCtrl tumor‐infiltrated neutrophils (Figure 6H), which was consistent with our initial observations (Figure 6D). Meanwhile, *Cdkn1a* and *Mif* were the most upregulated genes involved in the regulation of cell population proliferation in shNKX2‐1 tumor‐infiltrated neutrophils when compared to the shCtrl tumor‐infiltrated neutrophils (Figure 6H). The abundance of neutrophil‐positive regions was higher in shNKX2‐1 as compared to shCtrl tumors on the tumor cross‐sections utilized for Visium in situ capturing visualization. These regions markedly overexpressed genes that regulate cell communication and cell population proliferation (Figure 6I). In summary, our findings suggest that low expression of NKX2‐1 stimulates specific phenotypic properties in neutrophils, which potentially contribute to increased cancer progression through cell communication and cell proliferation. In other words, low levels of NKX2‐1 in cancer cells may create a pro‐tumor immune microenvironment, fostering malignant tumor development.

### 2.7 In Vivo Targeting of CXCLs/CXCR2 Signaling with SB225002 Reduces Tumor Growth and Neutrophil Infiltration

**Figure 7:**
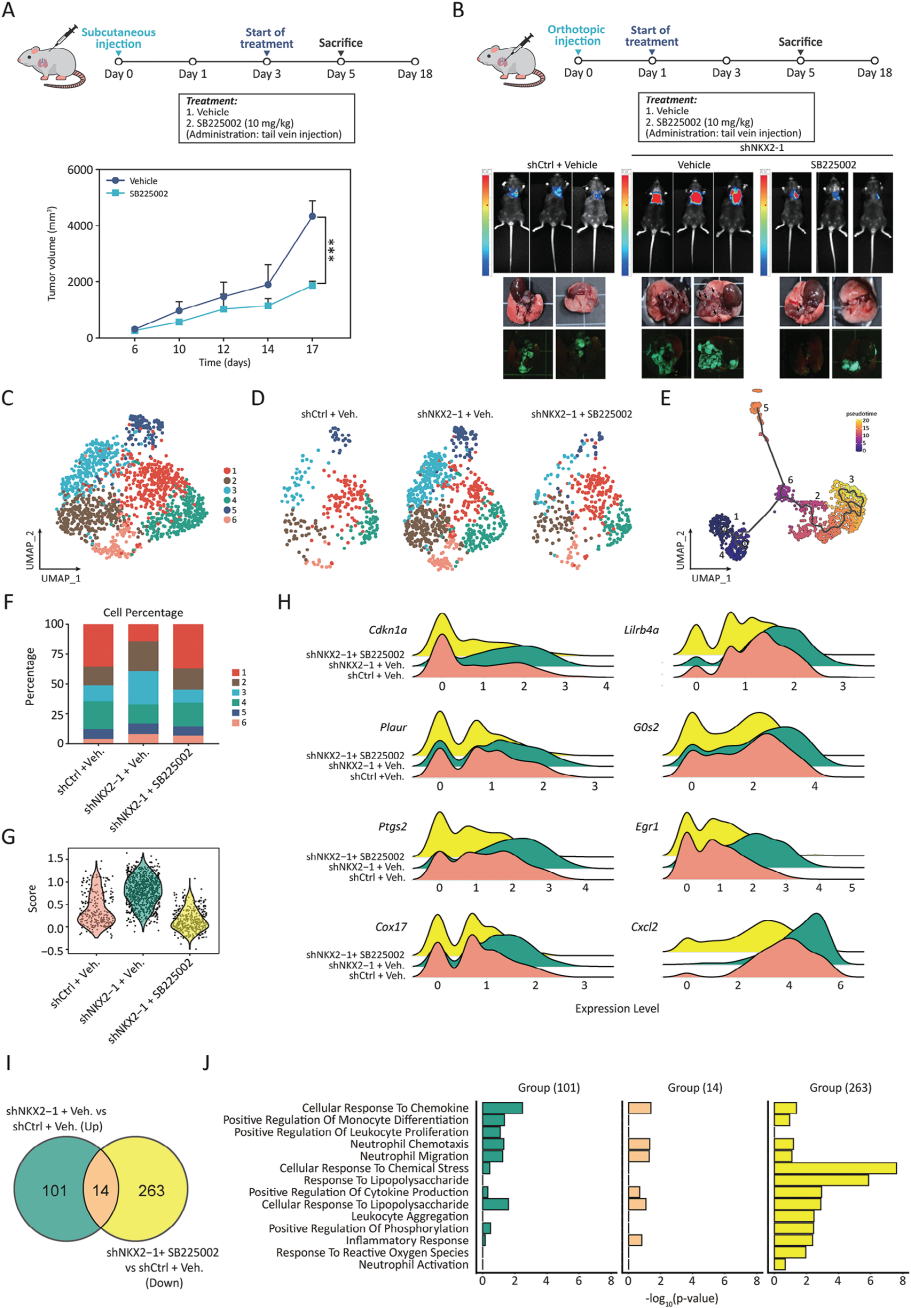
In vivo targeting of CXCLs/CXCR2 signaling with SB225002 reduces tumor growth and neutrophil infiltration. A) Subcutaneous mouse model. Top panel – schematic representation of the experimental design, bottom panel – tumor growth curve for 18 days following subcutaneous injection. Administration of SB225002 and vehicle control in the shNKX2‐1/LL2 tumor. Data are shown as means with SD error bars, N = 4, ***p < 0.001 (Student's t‐test). B) Orthotopic mouse model. Top panel – schematic representation of the experimental design, bottom panel – bioluminescent signal visualization (top), photographs and GFP fluorescence images of tumors (bottom) derived from shCtrl/LL2, shNKX2‐1/LL2, and shNKX2‐1/LL2 injected with or without (vehicle) SB225002 administration. C and D) Unsupervised clustering of scRNA‐seq data showing subdivision of infiltrated neutrophils into six distinct subpopulations (C) and in separate samples of shCtrl+vehicle, shNKX2‐1+vehicle, and shNKX2‐1+SB225002 cells (D). E) Pseudo‐time visualization showing the single‐cell lineage order based on the gene expression profile from scRNA‐seq analysis. F) Graphical representation of cell percentages according to neutrophil clusters with more than one read for the corresponding gene. G) Violin plot showing the gene expression score of upregulated cancer‐promoting genes, expressed in shCtrl+vehicle, shNKX2‐1+vehicle, and shNKX2‐1+SB225002 infiltrated neutrophils. H) Ridgeline plot visualization of the expression distributions of the indicated differentially expressed cancer‐promoting genes from cluster 3 across 3 samples (shCtrl+vehicle, shNKX2‐1+vehicle, and shNKX2‐1+SB225002 cells). I) The Venn diagram showing the numbers of NKX2‐1‐dependent genes involved in CXCR2‐mediated signaling. The left circle denotes the upregulated genes in shNKX2‐1+vehicle versus shCtrl+vehicle, and the right circle represents the downregulated genes in shNKX2‐1+SB225002 versus shNKX2‐1+vehicle samples. The overlapping genes are NKX2‐1‐regulated genes in the context of CXCR2‐mediated signaling. J) GO‐BP enrichment analysis of the indicated groups of 101, 14, and 263 genes shown in (I).

The identified NKX2‐1‐regulated CXC chemokines are known to share a common receptor, CXCR2. CXCR2 is a crucial chemokine receptor that facilitates neutrophil chemotaxis.^[^ ^40^ ^]^ The CXCLs/CXCR2 signaling is associated with increased cancer progression in LUAD^[^ ^15^ ^]^ aside from its significant role in recruiting neutrophils to inflamed sites.^[^ ^15^, ^41^, ^42^ ^]^ Previous reports have shown the potential of interfering with CXCLs/CXCR2 signaling to reduce tumor growth and enhance the efficiency of immunotherapy in different cancers.^[^ ^15^, ^41^, ^43^ ^]^ Since our findings are indicative of the modulatory role of NKX2‐1 in the LUAD TME, we sought to investigate whether targeting the CXCL/CXCR2 signaling pathway could suppress NKX2‐1‐low LUAD tumor growth and neutrophil infiltration. shCtrl/LL2 and shNKX2‐1/LL2 cells were orthotopically and subcutaneously injected with and without intravenous administration of SB225002. The experimental course for subcutaneous and orthotopic LUAD models was 18 days and 5 days, respectively, with SB225002 delivered every second day (**Figure** **7**A,B). The gross necropsy findings showed a remarkable tumor suppression by CXCR2 antagonism compared with the vehicle control recipient mice upon tail vein administration of SB225002 in the subcutaneous mouse model (Figure 7A). Monitoring the tumor growth in the orthotopic injection model by using the IVIS imaging system also showed the suppression of tumor growth by CXCR2 antagonism (Figure 7B). Altogether, our results demonstrate that low expression of NKX2‐1 fosters tumor growth, and targeting the CXCLs/CXCR2 axis with SB225002 mitigates the tumor growth induced by NKX2‐1 downregulation.

Next, we aimed to assess whether the CXCLs/CXCR2 signaling is essential for NKX2‐1‐dependent modulation of neutrophil recruitment and neutrophil infiltration. Chemotaxis assay demonstrated that SB225002 effectively blocked the migration of HL‐60 cells stimulated by shNKX2‐1/HCC827 and shNKX2‐1/H1279‐conditioned media (Figure S2F, Supporting Information). scRNA‐seq analysis was performed with a major focus on the neutrophil population following the inhibition of CXCR2 using SB225002. Specifically, mouse lungs were orthotopically implanted with shCtrl/LL2 and shNKX2‐1/LL2 cells, with or without subsequent administration of SB225002 (Figure 7B). The harvested tumors were dissociated into viable single cells, which were shown to pass standard scRNA‐seq quality control (Figure S3A–C, Supporting Information). Different neutrophil markers were used to identify the neutrophil population using the FindAllMarkers package (FigureS3D, Supporting Information), and UMAP analysis also indicated that only the neutrophil population expressed CXCR2 (Figure S3E, Supporting Information).

To distinguish between neutrophils and myeloid‐derived suppressor cells (MDSCs), given their similar phenotype and morphology,^[^ ^42^ ^]^ we validated the expression of CCR2 from our scRNA‐seq analysis. CCR2 is a typical marker of MDSCs.^[^ ^44^ ^]^ However, the result did not reveal the MDSC population. Instead, we observed an increased population of CCR2‐positive macrophages (Figure S3F, Supporting Information), characterized by high expression of *PgK1*, *Ccl9*, *Fcgr2b*, *Arg1*, and *Mt1* (Figure S3G, Supporting Information). Unsupervised clustering was performed on the neutrophil population which resulted in six clusters (Figure 7C,D). Each subpopulation was independently validated. The GO‐BP enrichment analysis was conducted on the upregulated genes derived from shNKX2‐1/LL2 cells as compared to shCtrl/LL2 cells within cluster 3. The analysis revealed a prevalence of processes such as neutrophil chemotaxis and migration, response to cytokine, and regulation of cell population proliferation, among others (Figure S3H, Supporting Information), which was consistent with the Visium in situ capturing profiling (Figure 6F). Indeed, NKX2‐1 knockdown increased the neutrophil population, while SB225002 treatment decreased it (Figure 7C,D). Additionally, pseudo‐time analysis was used to define the sequential lineage of single cells based on the gene expression profile obtained from the scRNA‐seq analysis (Figure 7E). The distribution pattern of cell percentages across six neutrophil clusters showed similarity between shCtrl/LL2‐derived tumors with vehicle control and SB225002‐treated shNKX2‐1/LL2 tumor, but it was different in shNKX2‐1/LL2‐derived tumors with vehicle control (Figure 7F). Unlike other clusters, cluster 3 exhibited the highest responsiveness to these experimental conditions. Its proportion significantly increased in shNKX2‐1 tumors as compared to shCtrl tumors. Conversely, shNKX2‐1 tumors treated with SB225002 showed the most pronounced decrease in proportion (Figure 7F). This observation suggests that blocking the CXCLs/CXCR2 signaling can reverse the effect of NKX2‐1 knockdown in terms of both the quantity and the landscape of infiltrated neutrophils. Notably, the neutrophil population of cluster 3 consistently exhibited an enrichment pattern across 3 different samples (shNKX2‐1/LL2 tumors with vehicle administration, shCtrl/LL2 tumors with vehicle administration, and shNKX2‐1/LL2 tumors with SB225002 administration). The result demonstrated significant enrichment in shNKX2‐1+vehicle, compared to the shCtrl+vehicle and shNKX2‐1+ SB225002 (Figure 7F).

To gain deeper insights into the potential role of the infiltrated neutrophils in tumor progression, we explored specific underlying cancer‐promoting genes within cluster 3. As illustrated by a violin plot, there was a notable upregulation of cancer‐promoting genes in shNKX2‐1/LL2+vehicle compared to both shCtrl/LL2+vehicle and shNKX2‐1/LL2+SB225002 (Figure 7G). We also explored the expression of the underlying cancer‐promoting genes within cluster 3; these genes included *Cdkn1a*, *Plaur*, *Ptgs2*, *Cox17*, *Lilrb4q*, *G0s2*, *Egr1*, and *Cxcl2*, with previous reports suggesting their implication in increasing cancer progression (Table S1, Supporting Information). As illustrated by a ridgeline plot, the expression distribution of these genes was highly similar in shCtrl/LL2+vehicle and shNKX2‐1/LL2+SB225002 samples, unlike in shNKX2‐1/LL2+vehicle sample, where the expression was shifted to more upregulated mode (Figure 7H). To identify the genes regulated by NKX2‐1 that may act in CXCLs/CXCR2 signaling‐dependent manner, we compared the list of genes upregulated in shNKX2‐1+vehicle compared to shCtrl+vehicle, i.e., genes under negative regulation by NKX2‐1, with the list of genes downregulated in shNKX2‐1+SB225002 compared to shNKX2‐1+vehicle, i.e., the genes whose action can be attributed to CXCLs/CXCR2 and abrogation of chemokine stimulation. The result revealed that 115 genes were associated with NKX2‐1 and cytokine stimulation, and 14 of those genes overlapped with the inhibition of CXCR2 signaling (Figure 7I). The 14 genes were *Wfdc17*, *Bsg*, *Tnfrsf26*, *Plin2*, *Hilpda*, *Hspa5*, *Ero1l*, *Egln3*, *Mif*, *Cxcl2*, *Bhlhe40*, *P4ha1*, *Nfkbiz* and *Chka*. GO‐BP analysis of these 14 genes indicated a decrease in the enrichment of cellular response to chemokine, neutrophil chemotaxis, neutrophil migration, positive regulation of cytokine production, cellular response to lipopolysaccharide, and inflammatory response as compared to the non‐overlapping genes in shNKX2‐1+vehicle versus shCtrl+vehicle and shNKX2‐1+SB225002 versus shCtrl+vehicle (Figure 7J). Altogether, our scRNA‐seq analysis demonstrated that the knockdown of NKX2‐1 promoted the infiltration of the neutrophil population with cancer‐promoting properties. This phenomenon could be counteracted by inhibiting the CXCLs/CXCR2 signaling with CXCR2 antagonists. Our data demonstrated the pivotal role of the CXCLs/CXCR2 signaling in NKX2‐1‐low tumor progression and cancer‐promoting neutrophil infiltration in LUAD. This suggests a potential method to control the malignant progression of NKX2‐1‐low LUAD tumors. Collectively, these findings emphasize that the CXCLs/CXCR2‐dependent mechanism is essential for tumor progression and neutrophil infiltration in NKX2‐1‐low LUAD.

### 2.8 Networks Engaged by NKX2‐1‐Low Tumor‐Activated Neutrophils in In Vitro and In Vivo Models

**Figure 8:**
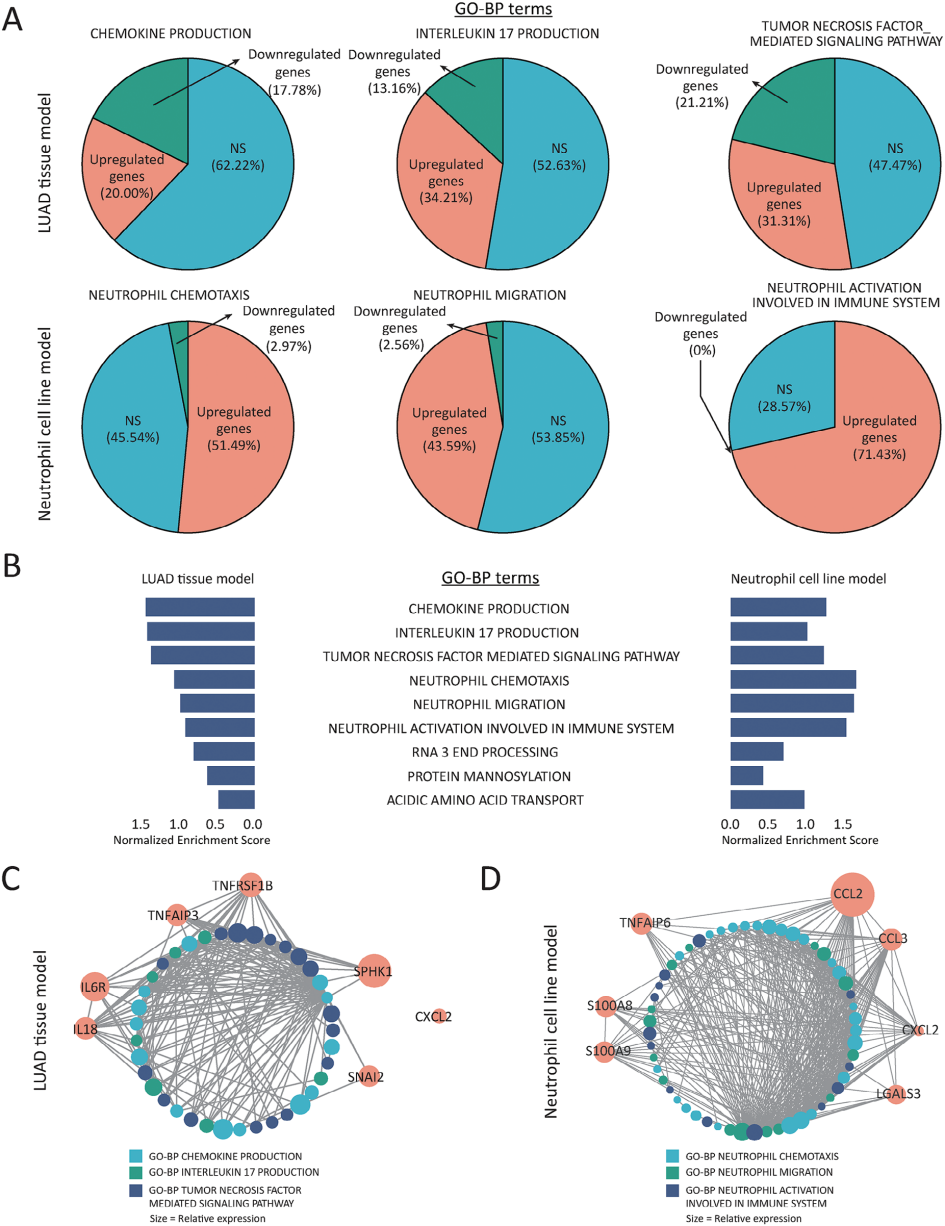
Networks engaged by NKX2‐1‐low tumor‐activated neutrophils in vitro and in vivo models. A) Pie chart representation showing the enriched GO‐BP terms with the upregulated gene percentage obtained from scRNA‐seq analysis of NKX2‐1‐low sample compared to the control group and RNA‐seq from HL‐60 cultured with the conditioned medium from shNKX2‐1/H1975 cells compared with shCtrl. B) Graphical representation of the enriched GO‐BP analysis from scRNA‐seq analysis of NKX2‐1‐low tumors compared to the control group and RNA‐seq from HL‐60 cultured with the conditioned medium from shNKX2‐1/H1975 cells compared with shCtrl. D and C) Network analysis showing different genes involved in the enriched GO‐BP analysis from scRNA‐seq analysis of NKX2‐1‐low sample compared to the control group (C) and RNA‐seq from HL‐60 cultured with conditioned medium from shNKX2‐1/H1975 cells compared with shCtrl (D).

In lung cancer, the activation of neutrophils in the TME introduces complexity to the inflamed environment by triggering additional mechanisms.^[^ ^27^ ^]^ Our observations indicate that the downregulation of NKX2‐1 promotes the recruitment and infiltration of neutrophils into LUAD tumors through the secretion of chemokines such as CXCL1, CXCL2, CXCL3, and CXCL5 into the TME (Figure 2, 3, 4), further fostering tumor‐promoting effects (Figure 6 and 7). Notably, among the CXC chemokines, CXCL1, CXCL2, and CXCL5 were reported to be involved in the paracrine network that mediates tumor progression and metastasis.^[^ ^45^ ^]^ To further evaluate the molecular events triggered by the tumor‐promoting neutrophils attracted by NKX2‐1‐low LUAD tumors, we analyzed both scRNA‐seq samples (NKX2‐1‐low tumors in comparison with the control) (Figure 3B) and RNA‐seq data from HL‐60 cells co‐cultured with shNKX2‐1/H1975‐conditioned medium compared to HL‐60 co‐cultured with shCtrl/H1975‐conditioned medium. This encompasses both in vivo and in vitro models, respectively (Figure 6A,B). GO‐BP enrichment analysis showed that neutrophils induced in NKX2‐1‐low LUAD tumor exhibited enrichment in chemokine production (20% upregulated genes), interleukin 17 (IL‐17) production (34.21% upregulated genes), and tumor necrosis factor‐mediated signaling pathway (31.31% upregulated genes) compared to the control tumor sample (**Figure** **8**A). Previous reports have shown the impact of IL‐17 production and tumor necrosis factor on tumor growth.^[^ ^45^ ^]^ This implies that the enrichment of tumor necrosis factor‐mediated signaling pathway and IL‐17 production in neutrophils underlies high inflammation, thereby promoting increased tumor growth. Additionally, HL‐60 cells cultured in shNKX2‐1/H1975‐conditioned medium showed the enrichment in neutrophil chemotaxis (51.49% upregulated genes), neutrophil migration (43.59% upregulated genes), and neutrophil activation involved in the immune system (71.43% upregulated genes) GO‐BP terms compared to the control group (Figure 8A). This suggests that neutrophils activated by NKX2‐1‐low cancer cells contribute to tumor progression by modulating the TME with tumor‐promoting molecules and inflammatory cells. Generally, both mice in vivo and human in vitro neutrophil models exhibited enrichment of the same GO‐BP terms, emphasizing NKX2‐1‐dependent effects (Figure 8B). A network analysis was conducted to further validate the genes involved in these enriched pathways. The results indicated a significant gene communication between *Tnfrsf1b*, *Tnfaip3*, *Sphk1*, *Snai2*, *Il6r*, and *Il18* in NKX2‐1‐low tumors (Figure 8C), while in HL‐60 cultured with shNKX2‐1/H1975 conditioned medium, a significant gene communication was revealed between *CXCL2*, *CCL2*, *CCL3*, *LGALS3*, *TNFAIP6*, *S100A8* and *S100A9* (Figure 8D). Altogether, our study elucidated the regulatory role of NKX2‐1 in the immune microenvironment of LUAD through the regulation of chemokine expression and secretion. This facilitates the recruitment and infiltration of tumor‐promoting neutrophils into the tumor, thereby contributing to tumor progression.

## 3 Discussion

Neutrophils are innate immune cells that play a crucial role in addressing infection and inflammation.^[^ ^8^ ^]^ In solid tumors, neutrophils are the most potent pro‐inflammatory cells, exhibiting a high intra‐tumor density that correlates with tumor metastasis and angiogenesis based on the stimulated cytokines and growth factors.^[^ ^35^ ^]^ The chemotactic recruitment of neutrophils is facilitated by chemokines such as CXCL1, CXCL2, CXCL3, CXCL5, CXCL6, CXCL7, and CXCL8, through the binding to their receptor CXCR2.^[^ ^39^ ^]^ Beyond their chemotactic role, CXC chemokines contribute to tumor cell proliferation, metastasis, chemoresistance, angiogenesis, invasion, and migration.^[^ ^45^ ^]^ Concurrently, the activation of CXCLs/CXCR2 signaling fosters tumor angiogenesis, progression, and chemoresistance.^[^ ^15^ ^]^ In lung cancer, lineage‐specific transcription factors regulate cancer cell identity by influencing the expression of other genes that shape the TME.^[^ ^19^ ^]^ However, how the infiltrated immune cells, particularly neutrophils, affect poorly differentiated LUAD tumors with low NKX2‐1 expression remains unclear. Here, we demonstrated that LUAD tumors with NKX2‐1 depletion exhibited increased infiltration of tumor‐promoting neutrophils via the activation of CXCLs/CXCR2 signaling.

Due to the dual role of NKX2‐1 as both a tumor suppressor and an oncogene, NKX2‐1 is not an ideal candidate for therapeutic targeting.^[^ ^46^ ^]^ In early‐stage NSCLC, particularly LUAD, the amplification of the NKX2‐1 gene locus was shown to significantly influence patient prognosis.^[^ ^47^, ^48^, ^49^, ^50^ ^]^ However, the decreased levels of NKX2‐1, or its loss, contribute to cancer progression.^[^ ^23^ ^]^ This is notably evident in the development of acquired resistance to EGFR tyrosine kinase inhibitors (TKIs), where the reduction or loss of NKX2‐1 prompts a transition from LUAD to lung SCC in EGFR‐mutant lung cancer.^[^ ^51^, ^52^, ^53^ ^]^ Hence, identifying targets associated with NKX2‐1 loss, which is primarily observed in more differentiated tumors (high‐grade tumors) with worse prognoses, has the potential to enhance patient survival. Here, we showed that the reduced expression of NKX2‐1 activated CXCLs/CXCR2 signaling, and targeting this pathway resulted in reduced tumor growth.

CXCR2 is a pivotal chemokine receptor with tumor‐promoting effects that facilitate the recruitment of leukocytes to inflamed tissues, resulting in tumor growth.^[^ ^40^ ^]^ In LUAD, CXCR2 is associated with tumor invasion, angiogenesis, and metastasis.^[^ ^15^, ^54^ ^]^ Additionally, the CXCLs/CXCR2 axis also plays a crucial role in promoting drug resistance in LUAD.^[^ ^15^ ^]^ In this context, we demonstrated that the downregulation of NKX2‐1 leads to the upregulation of CXCL1, CXCL2, CXCL3, and CXCL5, which are all ligands of CXCR2. IL‐17 is known to promote cancer progression by enhancing pro‐tumorigenic characteristics in lung cancer^[^ ^55^ ^]^ and influencing CXC chemokine expression in breast cancer.^[^ ^56^ ^]^ However, its precise role in the context of NKX2‐1‐low LUAD warrants further exploration. In this study, we demonstrated that NKX2‐1 can suppress the expression of CXC chemokines and trigger IL‐17 production (Figure 8), resulting in increased neutrophil recruitment. Previous reports have established that NKX2‐1 can directly bind to the proximal promoter regions or the various intragenic and intergenic regions of the regulated genes.^[^ ^57^ ^]^ On account of this observation, our ATAC‐seq analysis revealed that the downregulation of NKX2‐1 resulted in open chromatin structure at the promoter‐TSS of *CXCL1*, *CXCL2*, and *CXCL5* genes (Figure 5). Furthermore, we identified NKX2‐1‐binding motifs that could govern the regulation of these cytokine genes (Figure 5). This implies that the binding activity of NKX2‐1 at the regulatory elements of *CXCL1*, *CXCL2*, and *CXCL5* promotes the repressive states of these genes. Notably, this is consistent with the prior studies showing NKX2‐1 binding at the promoter and the first exon of murine *Cxcl5*.^[^ ^19^ ^]^ Consequently, targeting the CXCLs/CXCR2 axis could potentially enhance the clinical outcomes for NKX2‐1‐low LUAD patients.

To characterize the neutrophil population influenced by NKX2‐1 loss in LUAD malignancy, we applied scRNA‐seq and Visium in situ capturing to investigate the immune microenvironment heterogeneity in NKX2‐1‐low tumors. These novel tools offer unprecedented insight into cellular biology.^[^ ^58^, ^59^ ^]^ Our quantitative analyses revealed that NKX2‐1 downregulation led to increased neutrophil infiltration, a phenomenon mediated by CXC chemokines. Notably, this effect was abrogated by CXCR2 antagonism (Figure 7). While most studies often focus on neutrophil plasticity, cellular density, or maturation, the full potential of the genes expressed by neutrophils in the TME remains underexplored. It is crucial to highlight that our scRNA‐seq and Visium in situ capturing unveiled the expression of cancer‐promoting genes in the infiltrated neutrophils. This identification was achieved through unsupervised clustering, which revealed the broad gene expression patterns within the heterogenous neutrophil population. Notably, one of these clusters exhibited a significant expression of cancer‐promoting genes, encompassing *Cdkn1a*, *Plaur*, *Ptgs2*, *Cox17*, *Lilrb4q*, *G0s2*, *Egr1*, and *Cxcl2*. Several of these genes encode for secreted tumor‐promoting factors (Figure 7). Neutrophil infiltration correlates with tumor aggressiveness via tumor grades in human gliomas,^[^ ^28^ ^]^ and previous reports have also shown that the infiltration of neutrophils is related to an aggressive type of pancreatic tumor.^[^ ^60^ ^]^ Importantly, our study highlights the interplay among NKX2‐1, CXC chemokine signaling, and neutrophil infiltration. This correlates with tumor aggressiveness marked by high expression of tumor‐promoting genes by infiltrated neutrophils (Figure 3, 4, 5, 6, 7).

In summary, our study delineated the role of NKX2‐1 as a bona fide modulator of the immune tumor microenvironment of LUAD. Its depletion triggers the expression and secretion of CXC chemokines, thereby promoting increased recruitment and infiltration of neutrophils. These neutrophils exhibit cancer‐promoting properties with strong cell‐cell communication within the tumor, fostering a pro‐oncogenic TME. This ultimately drives increased tumor progression. Our findings reveal the role of the NKX2‐1/CXC chemokine signaling axis in orchestrating neutrophil infiltration and LUAD progression. Targeting CXCLs/CXCR2 signaling may be a viable treatment strategy to suppress tumorigenesis and improve the survival outcome of LUAD patients with NKX2‐1‐low malignant tumors.

## 4 Experimental Section

### Cell Culture

HCC827 (delE746_A750), H1975 (L858R/T790M), HCC827/GR, and H1975/AZDR lung cancer cells were obtained from Dr. Yu‐Ting Chou's laboratory (National Tsing Hua University, Taiwan). HCC827/GR and H1975/AZDR cells were initially established by treating HCC827 and H1975 cell lines with the increased concentrations of gefitinib and osimertinib for 6 months, and the surviving cells were pooled together and cultured.^[^ ^61^ ^]^ HL‐60 (CCL‐240) promyeoloblast cell line was obtained from the American Type Culture Collection (ATCC). These cells were cultured with RPMI‐1640 medium (Thermo Fisher Scientific, Waltham, MA, USA) supplemented with 10% fetal bovine serum (FBS), 100 mg mL^−1^ streptomycin, and 100 U mL^−1^ penicillin. Lewis lung carcinoma (LL2) cell line was obtained from Dr. Nien‐Jung Chen's laboratory (National Yang‐Ming Chiao Tung University). These cells were maintained in DMEM medium supplemented with 10% FBS, 100 mg mL^−1^ streptomycin, and 100 U mL^−1^ penicillin. All cells were maintained at 37 °C with 5% CO2 and were all tested negative for mycoplasma contamination. Gefitinib and osimertinib were purchased from Selleck Chemicals (Houston, TX, USA) and dissolved in dimethyl sulfoxide (DMSO; MP Biomedicals, Santa Ana, CA, USA) at a concentration of 10 mmol L^−1^.

### Animal Experiments

All animals used in this study were bred and maintained according to the Guidelines for Laboratory Animal Welfare in the Taipei Veterans General Hospital under the supervision of the Department of Medical Research of Taipei Veterans General Hospital (IACUC No. 2021–047 and 2022–097).

Female C57BL/6 mice were purchased from the Jackson Laboratory (Bar Harbor, ME, USA). Eight to ten‐week‐old mice were used in all the experiments. The mice were housed and maintained under specific‐pathogen‐free (SPF) conditions in an animal facility. In in vivo experiments, C57BL/6 mice and Lewis lung carcinoma (LL2) cell lines were used. CXCR2 inhibitor (SB225002) was dissolved in 1% DMSO, 20% polyethylene glycol 400, 5% Tween 80, and 74% ddH2O. SB225002 was administered at 10 mg k^−1^g by intraperitoneal injection every other day. Control groups received solvent (1% DMSO, 20% PEG 400, and 5% Tween 80). On day 0, LL2 cells expressing luciferase reporter and eGFP (shCtrl and shNKX2‐1) cells were collected and resuspended in PBS. For the subcutaneous tumor model, 100 µL cell suspension containing 5 × 10^5^ cells was injected subcutaneously into the flank region, while treatment started when the tumors were palpable. Mice were sacrificed on days 22–23. For the orthotopic lung cancer model, 20 µL cell suspension containing 5 × 10^5^ cells was injected through intrathoracic injection. The treatment started on day 1 and ended on day 5. Mice were sacrificed on day 7.

### Ethic Statements and Human Samples

The experimental procedures and protocols involving human samples were conducted according to the tenets of the Declaration of Helsinki and were approved by the Institutional Review Board of Taipei Veterans General Hospital (protocol no. 2020‐04‐009B and 2020‐10‐003B). Human tissue samples were obtained after taking informed consent from the patients.

### Plasmids, shRNAs, and Cell Transfection

shCtrl (pLKO.1), human shNKX2‐1 (TRCN0000020449_NM_0 03317 and TRCN0000020450_NM_0 03317), and mouse shNKX2‐1 (TRCN0000862665 and TRCN0000086267) were purchased from Academia Sinica RNAi core (Taipei, Taiwan). NKX2‐1 (pcDNA3.1(+) wt TTF‐1; 49 989) overexpression plasmid, and pHAGE PGK‐GFP‐IRES‐LUC‐W (46 793) plasmid were obtained from Addgene (Watertown, MA, USA). The lentivirus vector was co‐transfected with packaging and envelope plasmids (psPAX2 and pMD2G) into HEK 293T cells to obtain lentivirus particles. Viral supernatants were collected 72 h after transfection, followed by ultracentrifugation at 82700 g for 2 h. Cells were infected with lentivirus and 8 µg mL^−1^ polybrene (Sigma‐Aldrich, St Louis, MO, USA) according to the instructions of Addgene (http://www.addgene.org/). Subsequently, the cells were selected with puromycin (2 µg mL^−1^) to establish stable cell lines.

TransIT‐LT1 Transfection Reagent (Mirus Bio, Madison, WI, USA) was used for transient transfection. All procedures were conducted according to the manufacturer's guidelines. Plasmids used in this study are listed in Table S4 (Supporting Information). qRT‐PCR and immunoblotting were used to validate the knockdown efficiency by shRNAs.

### RNA Extraction and qRT‐PCR Analysis

The total RNA was extracted using RNeasy Mini Kit (QIAGEN, Hilden, Germany) and quantified by NanoDrop 2000 spectrophotometer (Thermo Fisher Scientific). According to the manufacturer's protocol, 1 µg of total RNA was subjected to first‐strand complementary DNA synthesis using the SuperScript III Reverse Transcriptase Kit (Thermo Fisher Scientific). qRT‐PCR reactions were performed using the SYBR Green kit in an ABI 7900 sequence detection system (Thermo Fisher Scientific) following the manufacturer's guidelines. The primers were designed using Primer Express Software v3.0.1 (Thermo Fisher Scientific) and are listed in Table S2 (Supporting Information). The specificity of all primers was computer‐tested using BLAST (National Center for Biotechnology Information, Bethesda, MD, USA) by homology search with the human or mouse genome and further confirmed by dissociation curve analysis. The relative expression of mRNA was determined by the 2^–ΔΔCT^ method and normalized to the endogenous expression of GAPDH mRNA.

### Western Blotting

The cells were lysed in RIPA lysis buffer (Beyotime Institute of Biotechnology, Haimen, China) containing proteinase and phosphatase inhibitor cocktail (Sigma‐Aldrich). The total protein concentration was determined by the Bradford assay (Bio‐Rad Laboratories, Hercules, CA, USA). Equal protein concentrations were resolved by SDS‐PAGE, transferred onto PVDF membranes (MilliporeSigma, Burlington, MA, USA), and blocked with 5% skimmed milk in Tris‐buffered saline with Tween 20. The membranes were incubated overnight at 4 °C with primary antibodies listed in Table S3 (Supporting Information). On the next day, the membranes were incubated with HRP‐conjugated secondary antibodies. Immunoblots were visualized using the Immobilon Western Chemiluminescent HRP Substrate (MilliporeSigma). The blots were tested with GAPDH or α‐tubulin antibodies to confirm equal protein loading.

### Human Chemokine Array

For secreted protein expression analysis, the Proteome Profiler Human Chemokine Array Kit (R&D Systems, Minneapolis, MN, USA; #ARY017) was used according to the manufacturer's protocol.

### RNA‐Seq Analysis

The purified RNA was used to prepare the sequencing library by TruSeq Stranded mRNA Library Prep Kit (Illumina, San Diego, CA, USA) following the manufacturer's instructions. Briefly, mRNA was purified from 1 µg of total RNA by oligo(dT)‐coupled magnetic beads and fragmented into small pieces under elevated temperature. The first‐strand cDNA was synthesized using reverse transcriptase and random primers. The adaptors were ligated after the generation of double‐stranded cDNA and adenylation of 3′ ends of DNA fragments. The products were enriched by PCR and purified with AMPure XP system (Beckman Coulter, Brea, CA, USA). The libraries were qualified by Qsep400 System (BiOptic Inc., New Taipei City, Taiwan) and quantified by Qubit 2.0 Fluorometer (Thermo Fisher Scientific). The qualified libraries were then sequenced on an Illumina NovaSeq 6000 platform with 150 bp paired‐end reads generated by Genomics, BioSci & Tech Co., (New Taipei City, Taiwan).

The low‐quality reads from the raw data were removed using the fastp (version 0.20.0 software). The filtered reads were aligned to the reference genomes using HISAT2 (version 2.1.0). The software featureCounts (v2.0.1) in the Subread package was applied to quantify the gene abundance. Differentially expressed genes were identified by DESeq2 (version 1.28.0)^[^ ^4^ ^]^ or EdgeR (version 3.36.0). The functional enrichment analysis of Gene Ontology (GO) terms was implemented in an R package clusterProfiler (version 4.0.0).

### Chromatin Immunoprecipitation Quantitative Real‐Time PCR (ChIP‐qPCR)

ChIP‐qPCR was performed using a High Cell Number Chromatin Immunoprecipitation kit (Diagenode, Denville, NJ, USA). 1 × 10^7^ cells mL^−1^ resuspended in PBS were processed according to the manufacturer's instructions. Chromatin was sonicated using a Bioruptor sonicator (Diagenode) according to the manufacturer's protocol and examined with an electrophoresis assay for shearing optimization. Enriched DNA was quantified by performing real‐time PCR using SYBR Green qPCR Master Mix (Thermo Fisher Scientific) in an ABI 7900 sequence detection system (Thermo Fisher Scientific) following the manufacturer's guidelines. The primers and antibodies used in ChIP‐qPCR assays are listed in Tables S2 and S3 (Supporting Information). The enrichment signal was normalized to input DNA.

### Assay for Transposase‐Accessible Chromatin with Sequencing (ATAC‐seq)

ATAC‐seq libraries were prepared by using an ATAC‐seq kit (Diagenode; #C01080001) following the manufacturer's instructions. In brief, 5 × 10^6^ H1975 (shCtrl and shNKX2‐1) cells were harvested, washed and the pellet was lysed. Nuclei were extracted, and tagmentation was performed according to the manufacturer's instructions. DNA was isolated by using a spin column and the transposase‐processed DNA fragments were amplified by using the 2X High‐Fidelity Master mix with 1 µL barcoded primers (Diagenode; #C01011035) for 13 cycles. AMPure XP beads (Beckman Coulter) were used to purify the DNA following the manufacturer's protocol. Qubit Flex Fluorometer was used to assess the DNA quality and integrity.

The FASTQ files were analyzed using the ENCODE ATAC‐seq pipeline for single‐end reads with default parameters. The genome fasta file was processed by the build_genome_data.sh script (supplied with the ATAC‐seq pipeline). The output mapping and peak files of each sample were further analyzed. The resulting files were visualized using the IGV viewer.^[^ ^62^ ^]^ MEME suites 5.5.4 were used to find the motif enrichment.

### Tissue Microarrays and Immunofluorescence

LUAD tissue microarray slides (TMAs) were obtained from US Biomax (Rockville, MD, USA). LC641 microarray panel contained 64 cases of LUAD, while the LC10013c microarray panel contained 48 cases of LUAD with matched adjacent normal lung tissues. TMA immunostaining was performed according to the manufacturer's protocol. The antibodies used are listed in Table S3 (Supporting Information). The scores of immunoreactivity patterns of all tissues from TMA were examined at the Department of Pathology, Taipei Veterans General Hospital, under the supervision of the Department of Medical Research and Education of Taipei Veterans General Hospital.

For immunofluorescence, a total of 1 × 10^5^ cells were plated onto a 35 mm dish. Following a 24‐h incubation period, the cells were fixed using 4% paraformaldehyde in PBS for 15 min and permeabilized with 0.5% TritonX‐100 in PBS for 15 min at room temperature. Subsequently, the cells were blocked with 10% BSA for 1 h at room temperature. After each step, the cells were washed thrice with PBS. The cells were then subjected to overnight incubation with NKX2‐1 antibody at 4 °C. Following this, the cells were exposed to a fluorescent‐labeled secondary antibody for 1 h along with DAPI (Sigma‐Aldrich) at room temperature. Finally, the cells were mounted using a mounting solution and examined under a microscope.

### Chemotaxis Assay and Co‐Culture Experiment

8 µm pore size FluoroBlok cell culture inserts (Corning Inc., Corning, NY, USA) were used to perform a chemotaxis transwell migration assay. HL‐60 cells were seeded in the transwell's upper chamber, and the medium from LUAD cells (HCC827 and H1975) was added to the lower chamber. After 16 h incubation, the migrated cells were fixed with methanol and stained with propidium iodide. The stained cells were viewed under a microscope and further quantified using ImageJ.

For the co‐culture experiment, the medium from shCtrl/H1975 or shNKX2‐1/H1975 cell culture was used to culture HL‐60 cells for 5 days. The medium was changed every other day.

### Flow Cytometry Analysis

Lungs containing tumor nodules were collected after mice were sacrificed. The mouse lung tissues were dissected and cut into small pieces. Dissected tissues were incubated with 1 mg mL^−1^ collagenase type I and DNase 1 in RPMI 1640 basic medium for 1 h at 37 °C. After incubation, the digested tissue was passed through a 70 µm cell strainer to improve cell dissociation.

Red Blood Cell Lysis Buffer (154 mM NH_4_Cl, 10 mM KHCO_3_, 0.1 mM EDTA, pH 7.4) was added to the single‐cell suspension to lyse red blood cells. The digested cells were washed 3 times and resuspended in PBS. 1 × 10^6^ cells mL^−1^ were stained with 1 µL fluorescence‐conjugated antibodies (BD Biosciences, Franklin Lakes, NJ, USA; dilution 1:100) for 30 min in 100 µL PBS at 4 °C. The cells were washed twice before flow cytometry analysis. The antibodies used are listed in Table S3 (Supporting Information), these are Pacific Blue‐labeled mouse anti‐human CD11b antibody, PerCP/Cyanine5.5‐labeled rat anti‐mouse CD170 (Siglec‐F), APC/Cyanine7‐ labeled rat anti‐mouse Ly‐6G, PerCP‐labeled rat anti‐mouse Ly‐6C, APC/Cyanine7‐labeled mouse anti‐human CD11b, Human Integrin alpha M/CD11b PE‐conjugated mouse anti‐mouse (R&D Systems; #FAB16991P), PE/Cyanine7‐labeled mouse anti‐mouse Ly‐6G, BV421‐labeled rat anti‐Mouse Siglec‐FData acquisition was performed on BD FACSCanto II System Flow Cytometer (BD Biosciences) and was analyzed by FlowJo Software.

### Single‐Cell RNA Sequencing (scRNA‐seq)

scRNA‐seq libraries of LL2 expressing luciferase reporter and eGFP cells (shCtrl, shNKX2‐1, and shNKX2‐1 treated with CXCR2 antagonist) were generated by using a Chromium Controller instrument (10x Genomics, Pleasanton, CA, USA) and Chromium Single Cell 3′ Reagent Kits v2 according to the manufacturer's instructions. scRNA‐seq reads were aligned to the mouse reference genome dataset and quantified using Cell Ranger version 6.1.0 (10x Genomics default pipeline. Raw count matrix files were imported into the R package Seurat version 4.1.1 for downstream processing. The package SoupX was used to remove ambient RNA contamination from raw scRNA‐seq data. Cells with a gene number of more than 500 and a mitochondrial gene proportion < 0.2 were selected for downstream analysis. The matrices used regularized negative binomial regression to normalize UMI count data by SCTransform (v2) and regress out the percentage of mitochondrial genes in each cell. We removed the doublet by Doubletfinder (estimate doublet ratio by 8 ‰ for every additional 1000 cells). After quality control and SCTransform (v2) normalization, the subsequent steps were based on the standard process of the Seurat package. 3000 feature genes were selected to integrate data. For the integrated data, RunPCA was used for data dimension reduction. The uniform manifold approximation and projection algorithm were used for visualizing the dimensionally reduced data by the RunUMAP function. Then we clustered cells by FindNeighbors and FindClusters functions based on the Leiden algorithm. FindConservedMarkers was used for cell type annotation based on gene markers. The FindMarkers function was used to find the differential gene expression between each group. The R package iTALK (0.1.0) was utilized to perform the crosstalk analysis between tumors and immune cells.

### Spatial Transcriptomics

Formalin‐fixed paraffin‐embedded (FFPE) samples passed the RNA quality control (DV200 > 50%). The tissue was prepared according to the Visium CytAssist Spatial Gene Expression for FFPE‐Tissue Preparation Guide (10x Genomics; #CG000618). The sequencing was performed by Genomics, BioSci & Tech Co. (New Taipei City, Taiwan). The Space Ranger pipeline v2.0.1 (10x Genomics) and the mm10‐2020‐A reference were used to process the FASTQ files. The accuracy criteria for shCtrl sample were: 2642 spots under the tissue, 80303 mean reads per spot, 8365 median genes per spot, 212160122 reads, 99.2% valid barcodes, 100% valid UMIs, and 26.7% sequencing saturation. For shNKX2‐1 sample: 1973 spots under the tissue, 89390 mean reads per spot, 8719 median genes per spot, 176365674 reads, 99.2% valid barcodes, 100% valid UMIs, 26.7% sequencing saturation. UMAP and violin plots were generated using Loupe Browser (10x Genomics). Trajectory analysis and pathway enrichment analysis were performed and plotted using Partek flow software (Partek Inc., Chesterfield, MO, USA).

### Statistical Analysis

Statistical analyses of data were presented as mean ± standard deviation by using Microsoft Excel and GraphPad Prism. Two‐tailed unpaired Student's t‐test was used for the two‐group comparison. One‐way ANOVA was used for multiple‐group comparisons. Survival curves were plotted using the Kaplan‐Meier method and assessed using a log‐rank test. The criterion for significance is set at *p* < 0.05.

### Ethical Approval

The collection of human tissue samples was conducted with informed consent in accordance with the International Ethical Guidelines for Biomedical Research Involving Human Subjects. The experimental procedures and protocols involving human samples were conducted according to the tenets of the Declaration of Helsinki and were approved by the Institutional Review Board of Taipei Veterans General Hospital (Protocol No. 2020‐04‐009B and 2020‐10‐003B).

## Conflict of Interest

The authors declare that they have no competing interests.

## Author Contributions

A.S.L. and L.‐J.C. contributed equally as the first authors. A.S.L., Y.‐T.C., M.‐L.W., S.‐H.C. performed formulation or evolution of overarching research goals and aims. A.S.L., J.C.‐Y.C., Y.‐C.C., C.M., and M.‐L.W. performed experiments, design, and execution. M.‐L.W., A.S.L., and M.‐L.W. performed animal experiments execution. P.‐H.T., L.‐J.C., K.‐H.L. performed bioinformatics analysis. P.‐K.H., Y.‐H.L., and Y.‐M.C. performed the provision of study materials. A.S.L., A.A.Y., M.‐L.W. wrote the first draft of the main manuscript text. M.‐L.W., Y.C., S.‐H.C., and Y.‐T.C. performed specific critical reviews, commentary, or revisions. A.A.Y.arranged and prepared figures. Y.‐C.C., M.‐L.W. manage and coordinate responsibility for the research activity planning and execution. M.‐L.W., S.‐H.C. performed oversight and leadership responsibility for the research activity planning and execution.

## Supporting information

Supporting Information

## Data Availability

The data that support the findings of this study are available on request from the corresponding author. The data are not publicly available due to privacy or ethical restrictions.
