# Supplementary material for: Neutrophils Recruited by NKX2‐1 Suppression via Activation of CXCLs/CXCR2 Axis Promote Lung Adenocarcinoma Progression
Source: Adv Sci (Weinh). 2024 Aug 7;11(38):2400370. doi: 10.1002/advs.202400370 (PMC11481344; doi:10.1002/advs.202400370)
Supplement: Supplementary file 1 — Supporting Information [file ADVS-11-2400370-s001.docx]

SUPPORTING INFORMATION


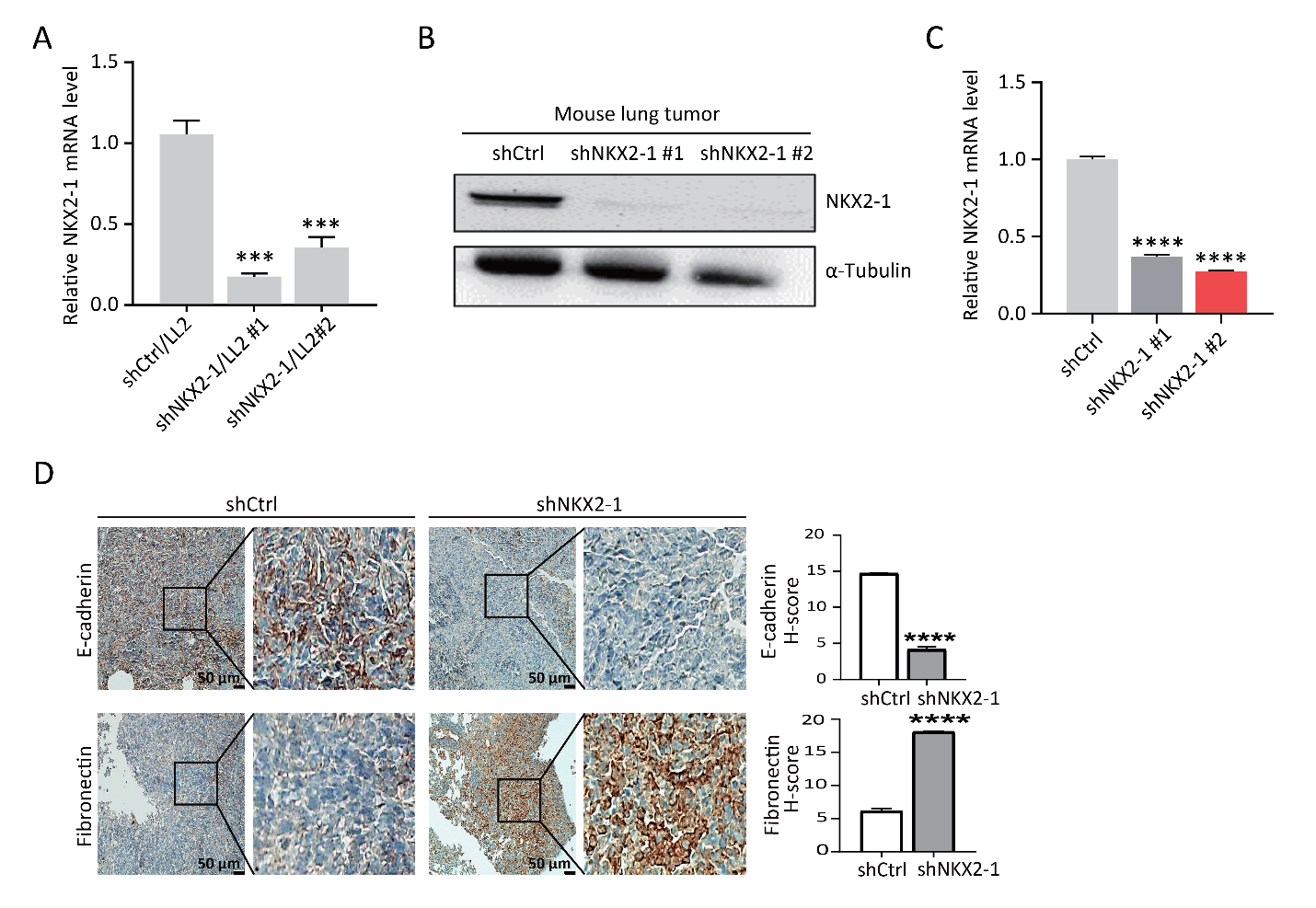


**Figure S1. The expression levels of NKX2-1 and EMT markers in LUAD samples. (A)** qRT-PCR analysis showing the expression levels of NKX2-1 in LL2 cells expressing luciferase reporter and eGFP transfected with NKX2-1-targeting shRNAs (shNKX2-1/LL2 #1 and shNKX2-1/LL2 #2). Mean fold changes (N=3) relative to shRNA control (shCtrl/LL2) are shown with SD error bars, *p<0.05, **p<0.01, ***p<0.001, ****p<0.0001, ns – not significant (Student’s t-test). **(B, C)** Immunoblotting **(B)** and qRT-PCR analysis **(C)** showing the expression level of NKX2-1 in excised mouse lung tumors from shCtrl/LL2, shNKX2-1/LL2 #1 and shNKX2-1/LL2 #2 cells. Mean fold changes (N=3) relative to shCtrl are shown with SD error bars, *p<0.05, **p<0.01, ****p<0.0001 (Student’s t-test). (**D)** IHC staining of EMT markers (E-cadherin and Fibronectin) performed on excised mouse tumor tissues derived from orthotopically injected shCtrl/LL2 and shNKX2-1/LL2 cells. Left panel: representative images. Right panel: H-score quantification. The data are presented as means ± SD error bars, ****p<0.0001 (Student’s t-test).


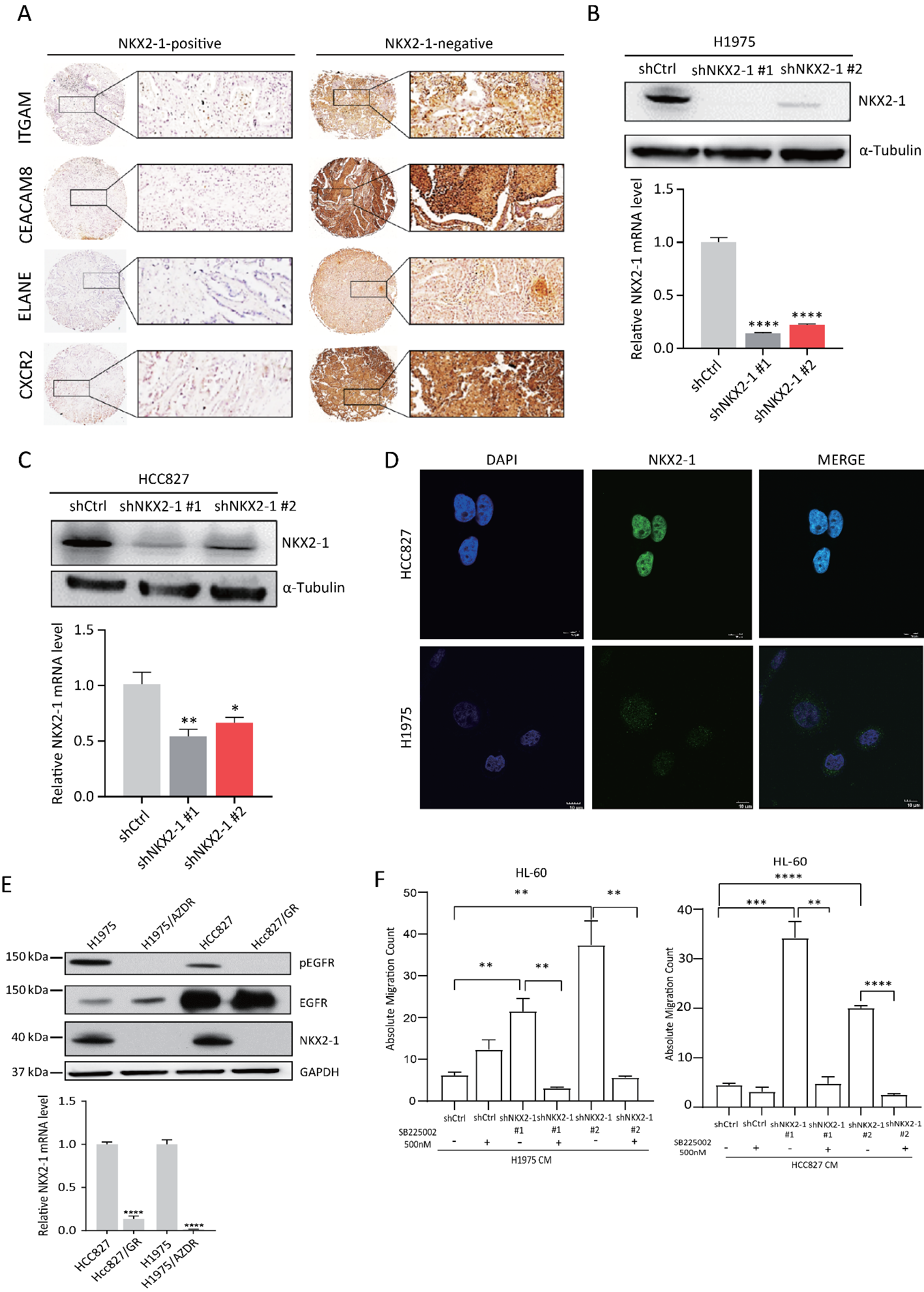


**Figure S2. Expression level of neutrophil markers and NKX2-1 in LUAD samples. (A)** The representative images of IHC staining performed on tumor tissue microarrays of NKX2-1-positive and negative LUAD tissues showing the expression of neutrophil markers (ITGAM, CEACAM8, ELANE, and CXCR2). **(B, C)** Immunoblotting and qRT-PCR analysis validating the expression of NKX2-1 in H1975 **(B)** and HCC827 **(C)** cells by transfected with NKX2-1-targeting shRNAs (shNKX2-1 #1 and shNKX2-1 #2). Top: immunoblotting, and bottom: qRT-PCR. Mean fold changes (N=3) relative to shCtrl are shown with SD error bars, *p<0.05, **p<0.01, ****p<0.0001 (Student’s t-test). (**D)** Immunofluorescence staining showing the localization of NKX2-1 in the nucleus of HCC827 and H1975 cells. **(E)** Immunoblotting and qRT-PCR analysis showing the expression levels of NKX2-1 in the indicated parental (H1975 and HCC827) and EGFR-TKI-resistant (H1975/AZDR and HCC827/GRC) LUAD cells. GAPDH – loading control. Mean fold changes (N=3) relative to parental cells are shown with SD error bars, **p<0.01, ***p<0.001, ****p<0.0001, ns – not significant (Student’s t-test). **(F)** Chemotaxis assay showing the migratory capacity of HL-60 cells in response to the conditioned media (CM) containing SB225002 derived from H1975 (left panel) and HCC827 (right panel) subjected to NKX2-1 knockdown. Mean numbers of migrated cells (N=3) are shown with SD error bars, **p<0.01, ***p<0.001, ****p<0.0001, ns – not significant (Student’s t-test).


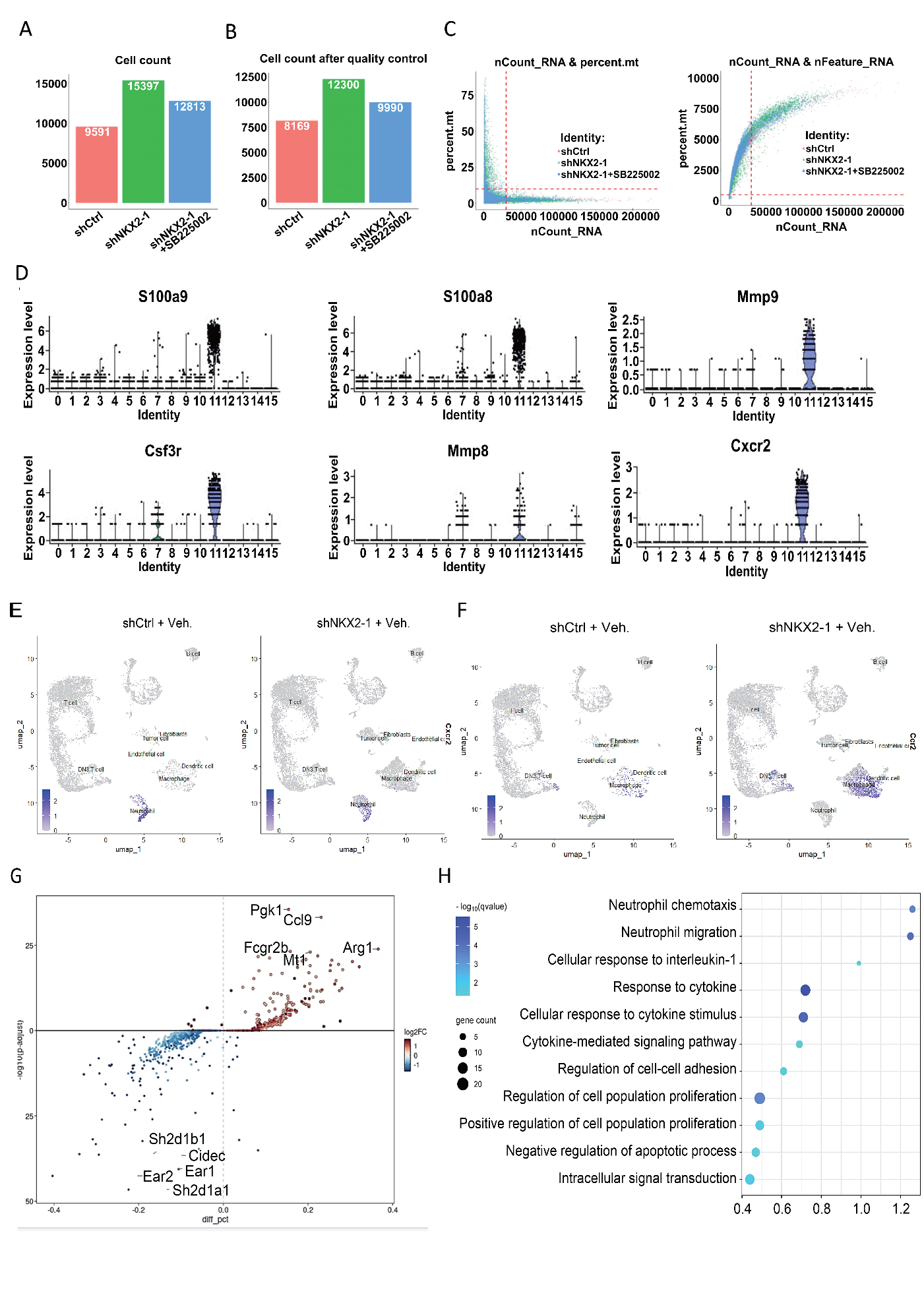


**Figure S3. scRNA-seq analysis quality control and characterization. (A, B)** Graphical representations for scRNA-seq quality control processing of shCtrl+vehicle, shNKX2-1+vehicle, and shNKX2-1+SB225002 raw data. **(C)** Scatter plots showing the correlation between UMI count and mitochondrial ratio, as well as the correlation between the RNA and UMI count**.** The red dotted lines indicated the filtered criteria cutoff. **(D)** Violin plot showing the neutrophil markers used to distinguish the neutrophil population from other cell types in scRNA-seq analysis. **(E, F)** UMAP analysis from scRNA-seq showing the expression of CXCR2 and CCR2 in shCtrl+vehicle, shNKX2-1+vehicle samples. **(G)** Differential expression gene (DEG) analysis from scRNA-seq analysis showing the most upregulated and downregulated genes in macrophages. **(H)** GO-BP enrichment analysis showing the biological processes in cluster 3 neutrophil population.

**Table S1:** List of cancer-promoting genes from scRNA-seq analysis

| **No.** | **Gene name** | **Gene Symbol** | **References** |
| --- | --- | --- | --- |
| 1. | Cyclin Dependent Kinase Inhibitor 1A | CDKN1A | [1-3] |
| 2. | Plasminogen activator, urokinase receptor | PLAUR | [4-6] |
| 3. | Prostaglandin-Endoperoxide Synthase 2 | PTGS2 | [7-9] |
| 4. | Cytochrome C Oxidase Copper Chaperone COX17 | COX17 | [10] |
| 5. | Leukocyte Immunoglobulin Like Receptor B4 | LILRB4 | [11-13] |
| 6. | G0/G1 Switch 2 | G0S2 | [14, 15] |
| 7. | Early growth response protein 1 | EGR1 | [16-18] |
| 8. | C-X-C Motif Chemokine Ligand 2 | CXCL2 | [19, 20] |

**REFERENCES**

1. Zaremba-Czogalla M, Hryniewicz-Jankowska A, Tabola R, Nienartowicz M, Stach K, Wierzbicki J, et al. A novel regulatory function of CDKN1A/p21 in TNFα-induced matrix metalloproteinase 9-dependent migration and invasion of triple-negative breast cancer cells. Cell Signal. 2018;47:27-36. Epub 20180326. doi: 10.1016/j.cellsig.2018.03.010. PubMed PMID: 29588220.

2. Wei CY, Tan QX, Zhu X, Qin QH, Zhu FB, Mo QG, et al. Expression of CDKN1A/p21 and TGFBR2 in breast cancer and their prognostic significance. Int J Clin Exp Pathol. 2015;8(11):14619-29. Epub 20151101. PubMed PMID: 26823785; PubMed Central PMCID: PMCPMC4713571.

3. Li Z, Qiu R, Qiu X, Tian T. SNHG6 Promotes Tumor Growth via Repression of P21 in Colorectal Cancer. Cell Physiol Biochem. 2018;49(2):463-78. Epub 20180829. doi: 10.1159/000492986. PubMed PMID: 30157475.

4. Xue A, Xue M, Jackson C, Smith RC. Suppression of urokinase plasminogen activator receptor inhibits proliferation and migration of pancreatic adenocarcinoma cells via regulation of ERK/p38 signaling. Int J Biochem Cell Biol. 2009;41(8-9):1731-8. Epub 20090321. doi: 10.1016/j.biocel.2009.03.004. PubMed PMID: 19433314.

5. LeBeau AM, Duriseti S, Murphy ST, Pepin F, Hann B, Gray JW, et al. Targeting uPAR with antagonistic recombinant human antibodies in aggressive breast cancer. Cancer Res. 2013;73(7):2070-81. Epub 20130211. doi: 10.1158/0008-5472.Can-12-3526. PubMed PMID: 23400595; PubMed Central PMCID: PMCPMC3618559.

6. Wang Z, Wang K, Gao X, Liu Z, Xing Z. Comprehensive analysis of the importance of PLAUR in the progression and immune microenvironment of renal clear cell carcinoma. PLoS One. 2022;17(6):e0269595. Epub 20220608. doi: 10.1371/journal.pone.0269595. PubMed PMID: 35675366; PubMed Central PMCID: PMCPMC9176830.

7. Saindane M, Rallabandi HR, Park KS, Heil A, Nam SE, Yoo YB, et al. Prognostic Significance of Prostaglandin-Endoperoxide Synthase-2 Expressions in Human Breast Carcinoma: A Multiomic Approach. Cancer Inform. 2020;19:1176935120969696. Epub 20201106. doi: 10.1177/1176935120969696. PubMed PMID: 33223820; PubMed Central PMCID: PMCPMC7656875.

8. Ogino S, Kirkner GJ, Nosho K, Irahara N, Kure S, Shima K, et al. Cyclooxygenase-2 expression is an independent predictor of poor prognosis in colon cancer. Clin Cancer Res. 2008;14(24):8221-7. doi: 10.1158/1078-0432.Ccr-08-1841. PubMed PMID: 19088039; PubMed Central PMCID: PMCPMC2679582.

9. Zahedi T, Hosseinzadeh Colagar A, Mahmoodzadeh H. PTGS2 Over-Expression: A Colorectal Carcinoma Initiator not an Invasive Factor. Rep Biochem Mol Biol. 2021;9(4):442-51. doi: 10.52547/rbmb.9.4.442. PubMed PMID: 33969138; PubMed Central PMCID: PMCPMC8068447.

10. Suzuki C, Daigo Y, Kikuchi T, Katagiri T, Nakamura Y. Identification of COX17 as a therapeutic target for non-small cell lung cancer. Cancer Res. 2003;63(21):7038-41. PubMed PMID: 14612491.

11. Kumata S, Notsuda H, Su MT, Saito-Koyama R, Tanaka R, Suzuki Y, et al. Prognostic impact of LILRB4 expression on tumor-infiltrating cells in resected non-small cell lung cancer. Thorac Cancer. 2023. Epub 20230608. doi: 10.1111/1759-7714.14991. PubMed PMID: 37290427.

12. Su MT, Kumata S, Endo S, Okada Y, Takai T. LILRB4 promotes tumor metastasis by regulating MDSCs and inhibiting miR-1 family miRNAs. Oncoimmunology. 2022;11(1):2060907. Epub 20220405. doi: 10.1080/2162402x.2022.2060907. PubMed PMID: 35402083; PubMed Central PMCID: PMCPMC8986222.

13. Sharma N, Atolagbe OT, Ge Z, Allison JP. LILRB4 suppresses immunity in solid tumors and is a potential target for immunotherapy. J Exp Med. 2021;218(7). Epub 20210511. doi: 10.1084/jem.20201811. PubMed PMID: 33974041; PubMed Central PMCID: PMCPMC8117208.

14. Nobeyama Y, Watanabe Y, Nakagawa H. Silencing of G0/G1 switch gene 2 in cutaneous squamous cell carcinoma. PLoS One. 2017;12(10):e0187047. Epub 20171026. doi: 10.1371/journal.pone.0187047. PubMed PMID: 29073263; PubMed Central PMCID: PMCPMC5658152.

15. Corbet AK, Bikorimana E, Boyd RI, Shokry D, Kries K, Gupta A, et al. G0S2 promotes antiestrogenic and pro-migratory responses in ER+ and ER- breast cancer cells. Transl Oncol. 2023;33:101676. Epub 20230420. doi: 10.1016/j.tranon.2023.101676. PubMed PMID: 37086619; PubMed Central PMCID: PMCPMC10214302.

16. Park SY, Kim JY, Lee SM, Chung JO, Lee KH, Jun CH, et al. Expression of early growth response gene-1 in precancerous lesions of gastric cancer. Oncol Lett. 2016;12(4):2710-5. Epub 20160805. doi: 10.3892/ol.2016.4962. PubMed PMID: 27698846; PubMed Central PMCID: PMCPMC5038579.

17. Kuo PL, Chen YH, Chen TC, Shen KH, Hsu YL. CXCL5/ENA78 increased cell migration and epithelial-to-mesenchymal transition of hormone-independent prostate cancer by early growth response-1/snail signaling pathway. J Cell Physiol. 2011;226(5):1224-31. doi: 10.1002/jcp.22445. PubMed PMID: 20945384.

18. Feng YH, Su YC, Lin SF, Lin PR, Wu CL, Tung CL, et al. Oct4 upregulates osteopontin via Egr1 and is associated with poor outcome in human lung cancer. BMC Cancer. 2019;19(1):791. Epub 20190809. doi: 10.1186/s12885-019-6014-5. PubMed PMID: 31399076; PubMed Central PMCID: PMCPMC6688208.

19. Zhang H, Ye YL, Li MX, Ye SB, Huang WR, Cai TT, et al. CXCL2/MIF-CXCR2 signaling promotes the recruitment of myeloid-derived suppressor cells and is correlated with prognosis in bladder cancer. Oncogene. 2017;36(15):2095-104. Epub 20161010. doi: 10.1038/onc.2016.367. PubMed PMID: 27721403.

20. Zhang F, Jiang J, Xu B, Xu Y, Wu C. Over-expression of CXCL2 is associated with poor prognosis in patients with ovarian cancer. Medicine (Baltimore). 2021;100(4):e24125. doi: 10.1097/md.0000000000024125. PubMed PMID: 33530204; PubMed Central PMCID: PMCPMC7850676.

**Table S2.** Primers for quantitative real-time PCR

| **Species** | **Gene name** |  | **Primers** |
| --- | --- | --- | --- |
| Mouse | NKX2-1 | forward | 5’- CATGTCGATGAGTCCAAAGC - 3’ |
|  |  | reverse | 5’- CTCCATGCCCACTTTCTTGT - 3’ |
|  | Cxcl1 | forward | 5’- TGCACCCAAACCGAAGTCAT - 3’ |
|  |  | reverse | 5’- ACTTGGGGACACCTTTTAGCA - 3’ |
|  | Cxcl2 | forward | 5’- CTGCCAAGGGTTGACTTCAAGA - 3’ |
|  |  | reverse | 5’- GCTTCAGGGTCAAGGCAAACT - 3’ |
|  | Cxcl3 | forward | 5’- CCAGACAGAAGTCATAGCCAC - 3’ |
|  |  | reverse | 5’- CTTCATCATGGTGAGGGGCTT - 3’ |
|  | Cxcl5 | forward | 5’- TGCCCTACGGTGGAAGTCAT - 3’ |
|  |  | reverse | 5’- GCGAGTGCATTCCGCTTA - 3’ |
|  | GAPDH | forward | 5’ - GGAGGAACCTGCCAAGTATG - 3’ |
|  |  | reverse | 5’ - TGGGAGTTGCTGTTGAAG - 3’ |
|  | EpCAM | forward | 5’- AAGAACCGACAAGGACACGG - 3’ |
|  |  | reverse | 5’ - TCTGATGGTCGTAGGGGCTT- 3’ |
|  | E-cadherin | forward | 5’- ATGTCCTGGGCAGAGTGAGA -3’ |
|  |  | reverse | 5’- TGGAGCTTTAGATGCCGCTT - 3’ |
|  | Vimentin | forward | 5’- TTCTCTGGCACGTCTTGACC - 3’ |
|  |  | reverse | 5’- GCTTGGAAACGTCCACATCG - 3’ |
|  | Fibronectin | forward | 5’- CAACCCTGGGTATGACACCG - 3’ |
|  |  | reverse | 5’- CCGCCTAAAGCCATGTTCCT - 3’ |
| Human | NKX2-1 | forward | 5’- AGCACACGACTCCGTTCTC - 3’ |
|  |  | reverse | 5’ - GCCCACTTTCTTGTAGCTTTCC - 3’ |
|  | CXCL1 | forward | 5’ - CACCCCAAGAACATCCAAAG - 3’ |
|  |  | reverse | 5’ - TAACTATGGGGGATGCAGGA - 3’ |
|  | CXCL2 | forward | 5’ - CACCTCAAGAACATCCAAAGTG - 3’ |
|  |  | reverse | 5’ - GATTTTCTTAACCATGGGCG - 3’ |
|  | CXCL3 | forward | 5’ - GAGCGTCCGTGGTCACTGAA - 3’ |
|  |  | reverse | 5’ - CCGGGGGACCTTACATTCAC - 3’ |
|  | CXCL5 | forward | 5’ - ACCACGCAAGGAGTTCATCC - 3’ |
|  |  | reverse | 5’ - GGGGCTTCTGGATCAAGACA - 3’ |
|  | EpCAM | forward | 5’ - GCAGGGTCTAAAAGCTGGTGTT - 3’ |
|  |  | reverse | 5’ - TCCCTATGCATCTCACCCATCT - 3’ |
|  | E-cadherin | forward | 5’ - ATTTTTCCCTCGACACCCGAT - 3’ |
|  |  | reverse | 5’ - TCCCAGGCGTAGACCAAGA - 3’ |
|  | Vimentin | forward | 5’ - GGCGAGGAGAGCAGGATTTC - 3’ |
|  |  | reverse | 5’ - AGTGGGTATCAACCAGAGGGA - 3’ |
|  | Fibronectin | forward | 5’ - ACCCCCACCAGCCTACTGAT - 3’ |
|  |  | reverse | 5’ - ACTTGCTCCCAGGCACAGTG - 3’ |
|  | ZEB1 | forward | 5’ - AGCAGTGAAAGAGAAGGG - 3’ |
|  |  | reverse | 5’ - GGTCCTCTTCAGGTGCCT - 3’ |
|  | ZEB2 | forward | 5’ - CCTCTGTAGATGGTCCAGAAGAA - 3’ |
|  |  | reverse | 5’- CTGTACCATTGTTAATTGCGGTC - 3’ |
| ChIP-qPCR | CXCL1 promoter | forward  reverse | 5’ - CCAGCCCCAACCATGCATAAAA - 3’  5’ - CTGTGAGAGGAGCGGAAGAGC - 3’ |
|  |  |  |  |
|  | Control-CXCL1 | forward  reverse | 5’ - ACTCTACCTGCACACTGTCCTA - 3’ |
|  |  |  | 5’ - ATGACTGGAGAACATTTGAAAACA  - 3’ |
|  | CXCL2 promoter | forward  reverse | 5’ - ATTCCCGGAGCTCCAGATCG - 3’  5’ - AAGACAGTCAGACCCGGACG - 3’ |
|  |  |  |  |
|  | Control-CXCL2 | forward | 5’ -TGCTACAACAATGTTCAAAGTCA - 3’ |
|  |  | reverse | 5’ – GCATTGGGATTTTATAGATTCTGGA- 3’ |
|  | CXCL5 promoter | forward | 5’- CATAGTGGTCAAGAGAGCG - 3’ |
|  |  | reverse | 5’ - GGGGAGAGATGAGTGTAGAT - 3’ |
|  | Control-CXCL5 | forward | 5’ - AAACATAACTTAGTGACAAGC - 3’ |
|  |  | reverse | 5’ - CTCTGTACGATTTAAGTAACA - 3’ |

**Table S3.** Antibody for Western blot, ChIP-qPCR, and Flow cytometry

| **Antibodies** | **Supplier** | **Species** | **Catalog No.** |
| --- | --- | --- | --- |
| TTF-1 (NKX2-1) | Cell Signaling Technology (CST) | Rb-Mono | 12373 |
| E-cadherin (24E10) | Cell Signaling Technology (CST) | Rb-Mono | 3195 |
| EpCAM | Cell Signaling Technology (CST) | Rb-Mono | 2626 |
| Vimentin | Sigma | Mouse-Mono | V6630 |
| ZEB1 | Cell Signaling Technology (CST) | Rb-Mono | 3396 |
| Phosphor-EGFR | Cell Signaling Technology (CST) | Rb-Mono | 3777 |
| EGFR | Cell Signaling Technology (CST) | Rb-Mono | 4267 |
| Alpha-tubulin | Sigma | Mouse-Mono | T6074 |
| CXCR2 | GeneTex | Mouse-Mono | GTX631667 |
| CD66b | Abcam | Rb-Poly | ab197678 |
| Neutrophil Elastase | Abcam | Rb-Poly | ab68672 |
| CD11b | Invitrogen | Rb-Mono | 14-0112-82 |
| PerCP/Cyanine5.5 CD170 (Siglec-F) | Biolegend | Mouse-Mono | 155525 |
| Pacific Blue™ CD11b | Biolegend | Mouse-Mono | 301316 |
| APC/Cyanine7 Ly-6G | Biolegend | Mouse-Mono | 127623 |
| PerCP Ly6-C | Biolegend | Mouse-Mono | 128027 |
| PE/Cyanine7, Ly-6G | Biolegend | Mouse-Mono | 127617 |
| BV421 Rat, Siglec-F | Biolegend | Mouse-Mono | 562681 |

**Table S4.** List of plasmids

| **Plasmids** | **Target sequence** | **Catalog No.** |
| --- | --- | --- |
| pcDNA3.1(+) wt TTF-1 | CDS region: NM_003317 | 49989 |
| p3xFLAG-Myc-CMV-26 | Vector backbone | Addgene |
| pHAGE PGK-GFP-IRES-LUC-W | Empty backbone | 46793 |
| pLKO.1 empty | Empty backbone | RNA icore |
| Human shRNA NKX2-1 pLKO.1 | CGCTTGTAAATACCAGGATTT | TRCN0000020449 |
| Human shRNA NKX2-1 pLKO.1 | CGGCATGAACATGAGCGGCAT | TRCN0000020450 |
| Mouse shRNA NKX2-1 pLKO.1 | CGGCATGAATATGAGTGGCAT | TRCN000086265 |
| Mouse shRNA NKX2-1 pLKO.1 | GTTCTCAGTGTCTGACATCTT | TRCN000008626 7 |
